# A cross-sectional and bioinformatics-based analysis: perirenal fat thickness as a superior predictor of kidney stone disease

**DOI:** 10.1186/s12944-025-02686-4

**Published:** 2025-08-29

**Authors:** Kaifeng Mao, Xiang Xu, Yifei Zhu, Fenwang Lin, Zhenquan Lu, Bingfeng Luo, Genggeng Wei, Yuan Yuan, Sucai Liao, Yaping Xing, Wenyan Huang, Ruidong Ji, Yige Pan, Zhenda Li, Junsheng Ye, Lin Xiong

**Affiliations:** 1 Division of Urology, Department of Surgery, The University of Hong Kong- Shenzhen Hospital, Shenzhen City, Guangdong Province China; 2 Department of Kidney Transplantation, Beijing Tsinghua Changgung Hospital, School of Clinical Medicine, Tsinghua Medicine, Tsinghua University, Beijing, 102218 China; 3 Department of Nursing, The University of Hong Kong-Shenzhen Hospital, Shenzhen City, Guangdong Province China; 4 Department of Thoracic Surgery, the University of Hong Kong-Shenzhen Hospital, Shenzhen City, Guangdong Province China

**Keywords:** Kidney stone, Obesity, Body mass index, Perirenal fat thickness, Inflammation

## Abstract

**Background:**

Kidney stone disease (KSD) is a growing global health concern, with obesity (OB) as a major risk factor linked to metabolic dysfunction and chronic inflammation. Although the common method for evaluating OB is body mass index (BMI), it is not specific enough when it comes to reflecting visceral fat. The perirenal fat thickness (PFT) might present better predictive capabilities. The goal of this research was to assess the clinical usefulness of PFT in the diagnosis of KSD and to clarify the molecular mechanisms connecting OB to KSD.

**Methods:**

Analysis was carried out on a retrospective cohort of 413 patients (265 having KSD and 148 controls). Abdominal computed tomography was used to measure PFT. Three machine-learning methods, weighted gene co-expression network analysis, and differential expression analysis were used to evaluate gene expression data for key gene identification. Internal and external datasets were used to develop and validate a diagnostic nomogram. Also, pathway enrichment analysis was carried out.

**Results:**

KSD patients exhibited greater PFT versus controls, with significantly enhanced diagnostic accuracy compared to BMI. Multivariate analysis confirmed PFT as an independent predictor of KSD (OR = 1.20, *P* < 0.001). Eight genes that are differentially expressed in relation to OB were identified, among which FAM20A and DHRS9 were found to be central hub genes. The nomogram exhibited a high level of predictive accuracy. Analysis of enrichment pointed to the IL-6/JAK/STAT3 and TNF-α/NF-κB signaling pathways in the connection between perirenal fat and KSD.

**Conclusions:**

PFT serves as a practical and dependable marker for the risk of KSD. It is superior to BMI and can be conveniently incorporated into routine clinical practice. Stone formation may be linked to perirenal fat by FAM20A and DHRS9 via inflammatory pathways, which provides potential targets for the management of OB-related KSD.

**Supplementary Information:**

The online version contains supplementary material available at 10.1186/s12944-025-02686-4.

## Introduction

The formation of crystalline deposits within the renal system characterizes kidney stone disease (KSD), which is a common urological condition [1, 2]. It brings about a great global health problem and also causes a large amount of economic and healthcare burdens [3, 4]. In China, the rate of a certain renal system-related disease is notably higher than the estimated global prevalence range [5].

Globally, obesity (OB) has become a crucial health problem at the same time. A substantial global adult population was classified as obese in 2015, with projections suggesting that nearly half of the adult population in the United States will be obese (BMI ≥ 30) by 2030 [6, 7]. Excessive body weight is only one aspect of OB; it is also closely tied to metabolic dysfunctions like insulin resistance, dyslipidemia, and chronic inflammation [8]. A large number of studies have constantly shown that OB, especially when combined with metabolic dysfunction and chronic inflammation, greatly elevates the risk of KSD [8–11].

Although body mass index (BMI) is still the traditional method for evaluating OB, it has been criticized because it can’t distinguish between visceral and subcutaneous adiposity [12]. Perirenal fat, which is next to the kidneys and is a metabolically active adipose depot that secretes adipokines and pro-inflammatory cytokines [13, 14], is of special interest. Some recent research indicates that the thickness of perirenal fat (PFT) could be a more precise predictor of metabolic disorders related to OB and their complications compared to BMI [15–17].

While existing literature indicates a connection between perirenal fat and KSD, a great deal of the current research depends on advanced 3D imaging technologies and intricate analytical methods, which restricts their use in routine clinical work [5, 18]. On the contrary, standard computed tomography (CT) imaging, which is often used in clinical situations, can be used to measure PFT easily and dependably. This makes it a useful and effective measure for incorporating into regular diagnostic procedures. For clinicians with a busy schedule, PFT is a convenient and time-saving substitute for assessing KSD risk in obese patients without requiring specialized devices.

This research measured the association between PFT and KSD risk. In addition, it aimed to find out possible molecular biomarkers and clarified the mechanistic paths connecting the fat around the kidney to stone formation, thus offering understanding for clinical decision-making and personalized risk evaluation strategies.

## Methods

### The design of the study and the patient group

Formal approval for this research was granted by the Ethics Committee of the University of Hong Kong-Shenzhen Hospital (Approval No. [2025]041). In a single institute, a retrospective analysis was carried out on 840 patients who were admitted to the Urology Department between January and December in 2020. Based on kidney stone status, participants were categorized into two groups: a KSD group (*n* = 265; unilateral kidney stone-positive) and a control group (*n* = 148; bilateral kidney stone-negative).

**Fig. 1:**
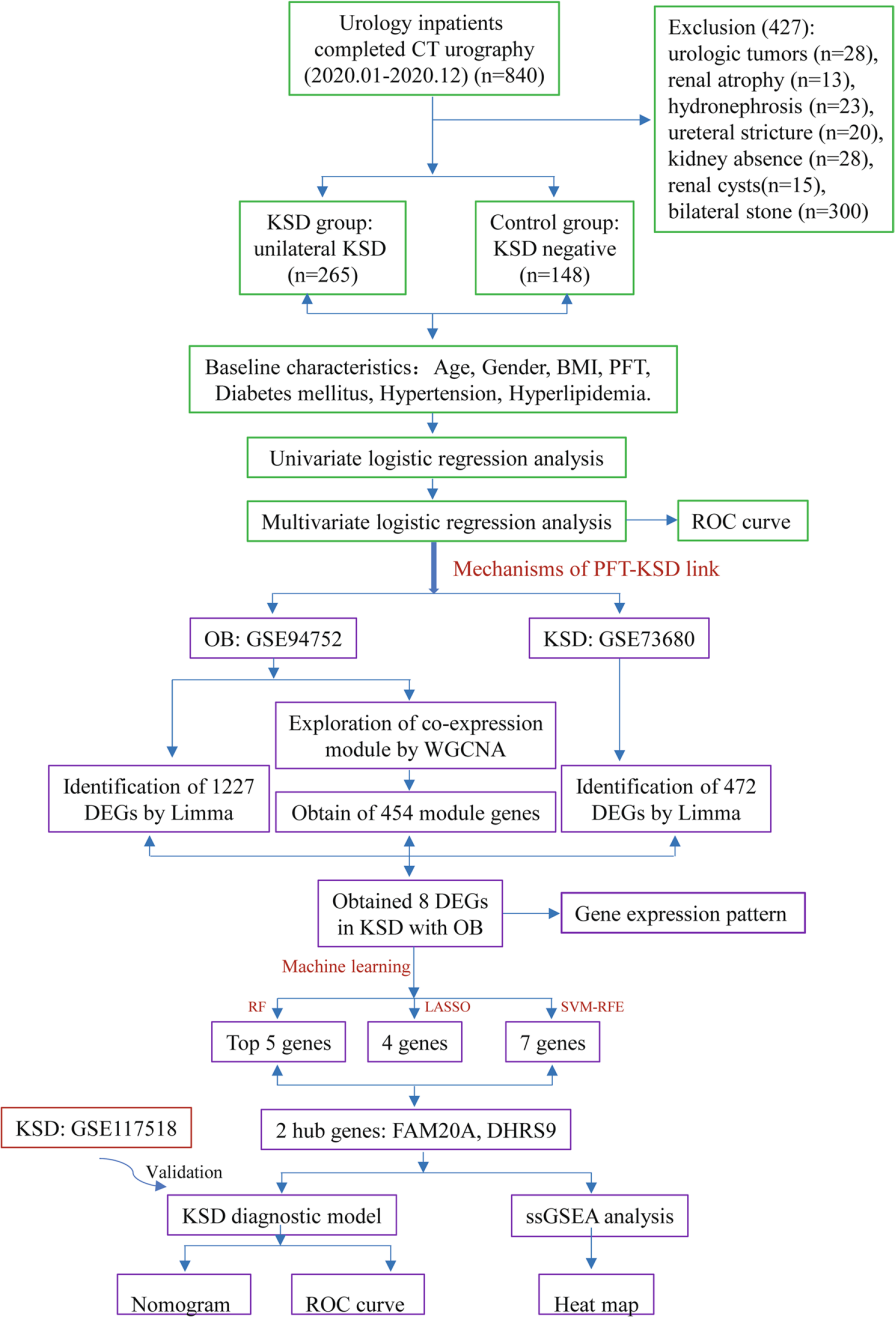
Flowchart of this study. OB: obesity; PFT: perirenal fat thickness; KSD: kidney stone disease; BMI: body mass index; WGCNA: Weighted Gene Co-expression Network Analysis; DEGs: differentially expressed genes; ORDEGs: obesity-related Differentially expressed genes; RF: random forest; LASSO: least absolute shrinkage and selection operator; SVM-RFE: support vector machine-based feature selection technique; ROC: receiver operating characteristic

The exclusion criteria included urologic tumors (*n* = 28), renal atrophy (*n* = 13), moderate-to-severe hydronephrosis (*n* = 23), ureteral strictures (*n* = 20), unilateral renal agenesis (*n* = 28), renal cysts (*n* = 15), and bilateral kidney stones (*n* = 300). Figure 1 schematizes the methodological workflow.

Baseline characteristics included age, gender, BMI, and history of: (1) diabetes mellitus (elevated fasting glucose ≥ 7.0 mmol/L or HbA1c ≥ 6.5%); (2) hypertension (systolic blood pressure [SBP] ≥ 140 mmHg and/or diastolic blood pressure [DBP] ≥ 90 mmHg on ≥ 2 separate occasions); and (3) hyperlipidemia (total cholesterol ≥ 5.2 mmol/L and/or LDL-C ≥ 3.4 mmol/L and/or triglycerides ≥ 1.7 mmol/L).

### The collection of computed tomography (CT) image data and the measurement of the perirenal fat thickness (PFT)

**Fig. 2:**
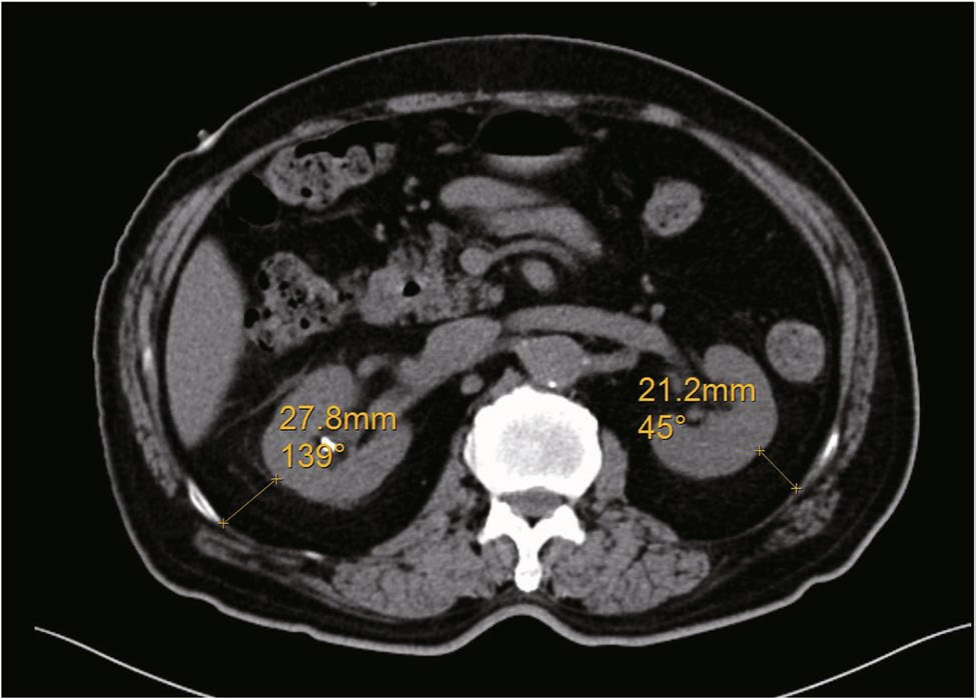
PFT at the renal hilum level can be evaluated by means of transverse CT imaging. The yellow line runs from the renal capsule to the sidewall. PFT: perinephric adipose tissue; CT: computed tomography

Two scanner platforms were used to perform standard abdominal CT scans on all participants in the supine position: (1) The GE Lightspeed Ultra 16 (64 - detector rows, 1.25 mm slice thickness, pitch 1.5, tube voltage 120 kVP, and mAs in the range of 100–200 [automated modulation]); (2) The Siemens Somatom Definition AS (1.0 mm slice thickness, pitch 1.2, tube voltage 120 kVP, and mAs 100–200). High - resolution algorithms were used by both scanner platforms to generate multiplanar reconstructions (with a thickness of 3 mm and intervals of 3 mm). Axial CT sections were used to quantify PFT as per Mayo Clinic standards [19], with the measurement of the horizontal distance between the renal parenchymal border and the posterior rectus sheath along the renal venous axis (Fig. 2). All measurements were carried out by two radiologists who were certified and blinded to all clinical information. For the subjects in the control group, the largest value of either the left or right PFT was chosen for analysis.

### The acquisition of microarray data

Microarray datasets associated with OB and KSD were downloaded from the Gene Expression Omnibus (GEO; http://www.ncbi.nlm.nih.gov/geo): GSE94752 (OB discovery cohort), GSE73680 (KSD discovery cohort), and GSE117518 (KSD validation cohort). These data formed the basis for subsequent transcriptomic and machine-learning analyses.

### Co-expression gene modules analysis

To identify co-expressed gene modules and evaluate their correlations with clinical traits, the weighted gene co-expression network analysis (WGCNA) method was utilized [20, 21]. A subset of highly variable genes was selected for network construction. A soft-thresholding power of β = 2 was selected to satisfy scale-free topology criteria (scale-free R² >0.8). A signed topological overlap matrix (TOM) was then calculated, followed by dynamic tree-cutting (minimum module size = 100 genes, merge cut height = 0.25), yielding five discrete modules. Module eigengenes were correlated with OB status using Pearson’s test; modules surpassing |r| > 0.5 and FDR < 0.05 were considered trait-relevant. Within these modules, genes exhibiting module membership (MM) > 0.8 and gene significance (GS) > 0.5 were retained as hub candidates for subsequent integration.

### The identification of genes with differential expression (DEGs)

Differentially expressed genes (DEGs) were identified by using the limma package (v4.4.3) in R. An empirical Bayes moderation-incorporated linear model was applied. Genes with *P* < 0.05 and absolute log fold change > 0.5 were considered statistically significant. Venn diagrams were used to visualize the genes obtained from the intersecting of DEGs lists of GSE94752 and GSE73680 and the key WGCNA modules to identify OB-related DEGs (ORDEGs). Volcano plots in ggplot2 and heatmaps in pheatmap were created for visualizations.

### Algorithms in machine learning

Hub genes related to diseases can be effectively identified by machine-learning methods. For biomarker screening, several well-known machine learning algorithms are widely used, like random forest (RF), least absolute shrinkage and selection operator (LASSO), and support vector machine-based feature selection technique (SVM-RFE) as mentioned in the previous research [22]. Variable filtration is carried out by LASSO regression to reduce overfitting [23]. When it comes to handling high-dimensional datasets, random forest (RF) has an advantage, which allows for strong predictive modeling and precise estimation of variable importance [24]. The SVM-RFE method was used to gradually remove the least significant variables, and finally optimize the subset of crucial genes for better classification performance [22]. The hub ORDEGs in the KSD were determined as the overlapping genes found by these three machine learning algorithms, and they were shown in a Venn diagram.

### The construction and validation of nomogram

Using the rms package in R, diagnostic nomograms for OB-related KSD were built [25]. There were two main scales in the nomogram: one was a “points” scale, which gave individual scores to each gene, and the other was a “total points” scale, indicating the cumulative contribution of all genes. Calibration curves and decision curve analysis (DCA) were utilized to appraise the clinical prediction efficiency of the nomogram. Also, the discriminative capacity of the nomogram was evaluated by means of receiver operating characteristic (ROC) curve analysis. A value of area under the curve (AUC) more than 0.7 was considered clinically significant, which showed strong predictive power [26]. The generalizability of the nomogram was evaluated by using the GSE117518 dataset for external validation.

### Analysis of pathway enrichment

Single-sample gene set enrichment analysis (ssGSEA) was performed using the GSVA package (v1.48.3) and MSigDB hallmark gene sets (h.all.v2024.1.Hs.symbols.gmt) [27]. Significant biomarker-pathway correlations (Pearson, *P* < 0.05) were identified via heatmap visualization. Scatter plots and regression analysis further highlighting the top associations.

### Statistical analysis

R software (version 4.3.2), GraphPad Prism (version 8.0.2), and SPSS (version 25.0) were utilized for statistical analyses. When it comes to continuous variables, Student’s t test was used to make comparisons between two groups. Either the chi-square (χ^2^) test or Fisher’s exact test, depending on the data distribution, was used to analyze categorical variables. Pearson’s correlation coefficient was utilized to analyze the relationship between PFT results and BMI. To assess the clinical predictors of KSD, univariate and multivariate regression analyses were utilized. A two-tailed *P* < 0.05 was considered statistically significant.

## Results

### Clinical characteristics at the baseline

A total of 265 patients with KSD and 148 controls were included in this study. Both groups had comparable mean ages (54.4 vs. 53.6 years, *P* = 0.646). However, a significantly higher proportion of males was observed in the KSD group compared to controls (77.7% vs. 60.1%, *P* = 0.029), and patients with KSD exhibited a notably higher BMI (24.48 vs. 22.45 kg/m2, *P* < 0.001). Importantly, PFT was significantly elevated in the KSD group (20.35 vs. 10.98 mm, *P* < 0.001), reinforcing its potential association with stone formation. Additionally, the prevalence of diabetes (24.9% vs. 16.2%, *P* = 0.040) and hyperlipidemia (23.8% vs. 10.8%, *P* = 0.001) was significantly higher in the KSD group. However, no significant difference was found in hypertension prevalence (34.0% vs. 27.7%, *P* = 0.19). A summary of these clinical characteristics can be found in Table 2.

**Table 1:** The study contains information regarding the datasets

GSE series	Array type	Species	Source types	Number	Utilization
GSE94752	GPL11532	*Homo sapiens*	Abdominal subcutaneous adipocytes	30 obesity vs. 9 control adipocytes	1) OB associated DEGs extraction;2) Key module gene mining via WGCNA
GSE73680	GPL17077	*Homo sapiens*	Human kidney papillary tissue biopsies	29 plaque vs. 6 normal renal papillary samples	(1) KSD associated DEGs extraction; (2) Hub-gene mining via ML; (3) Nomogram building
GSE117518	GPL21827	*Homo sapiens*	Human kidney papillary tissue biopsies	3 plaque vs. 3 normal renal papillary samples	Validating the nomogram model

Note: *OB* Obesity, *KSD* for kidney stone disease, *WGCNA* Weighted Gene Coexpression Network Analysis, *DEG* Differentially expressed gene, *ML* Machine learning

**Table 2:** Baseline demographic of the study cohort (*n* = 413)

Variable	KSD group (*n* = 265)	Control group (*n* = 148)	*P*
Age (years)	54.38 ± 15.27	53.61 ± 17.69	0.646
Gender, n (%)			0.029
Male	206 (77.7%)	89 (60.1%)	
Female	59 (22.3%)	59 (39.9%)	
BMI (kg/m^2^)	24.48 ± 3.33	22.45 ± 3.61	< 0.001
PFT (mm)	20.35 ± 7.69	10.98 ± 6.77	< 0.001
History of diabetes			0.04
Yes, n (%)	66 (24.9%)	24 (16.2%)	
No, n (%)	199 (75.1%)	124 (83.8%)	
History of hypertension			0.19
Yes, n (%)	90 (34.0%)	41 (27.7%)	
No, n (%)	175 (66.0%)	107 (72.3%)	
History of hyperlipidemia			0.001
Yes, n (%)	63 (23.8%)	16 (10.8%)	
No, n (%)	202 (76.2%)	132 (89.2%)	

Note: *KSD* Kidney stone disease, *BMI* Body mass index, *PFT* Perirenal fat thickness

### The causal link between OB and KSD

**Fig. 3:**
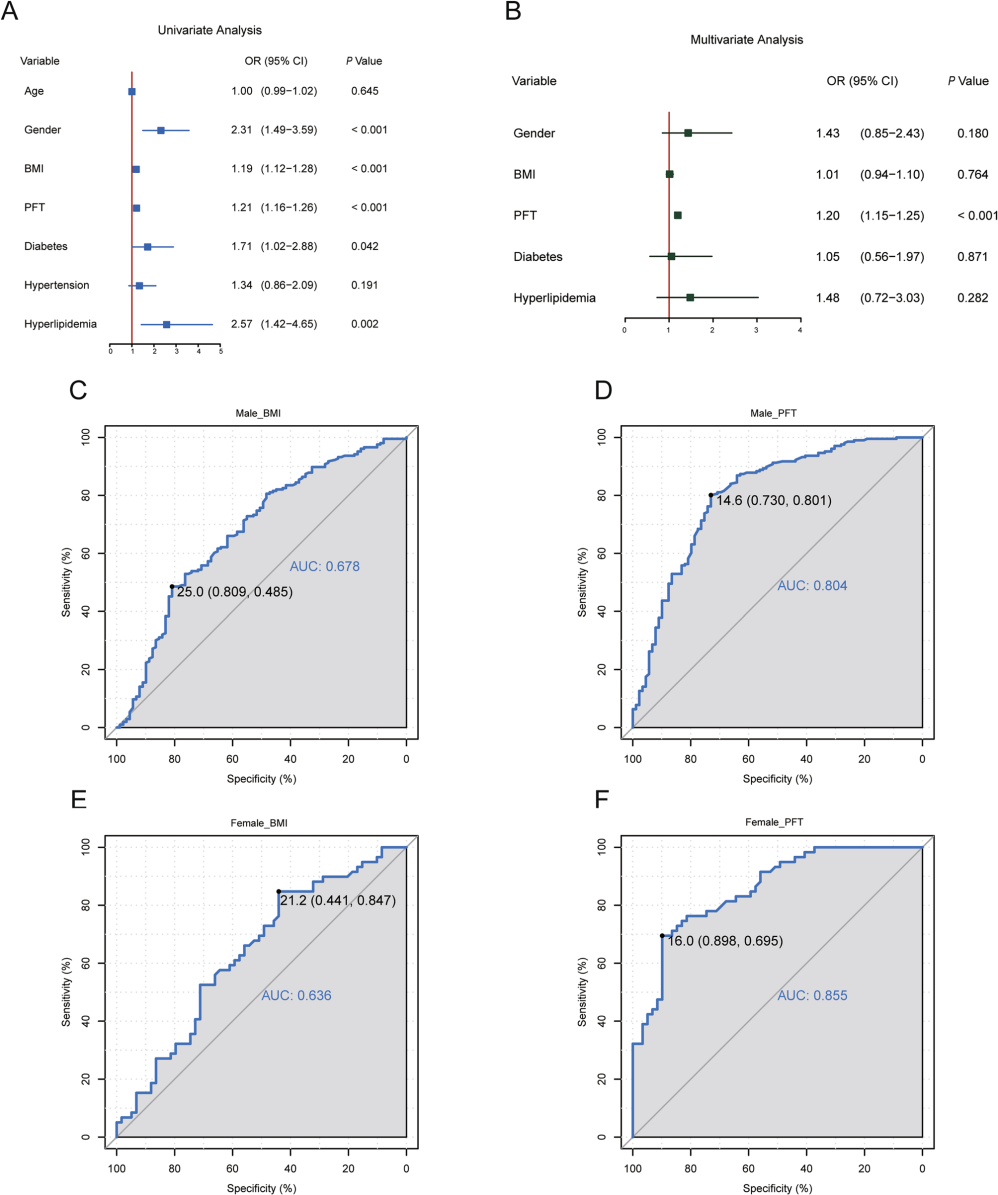
The connection between OB and KSD risk. Univariate (**A**) and multivariate (**B**) logistic regression analyses of KSD. ROC curves for evaluating BMI (**C**) and PFT (**D**) for KSD diagnosis in males and BMI (**E**) and PFT (**F**) for KSD diagnosis in females. The values of AUC (95%CI) represent the area under the curve with 95% confidence intervals. OB: obesity; KSD: kidney stone disease; PFT: Perirenal fat thickness; BMI: Body mass index; ROC: Receiver operating characteristic; AUC: Area under the curve

Mendelian randomization (MR) analysis was carried out first (Supplementary Materials 1 and 2) in order to evaluate whether OB has a causal effect on the risk of KSD. The findings of the MR analysis hinted at a possible causal connection between OB and the emergence of KSD. Clinical data were used to further investigate this association. Figure 3A showed that in univariate logistic regression, gender, BMI, PFT, diabetes, and hyperlipidemia were identified as important risk factors for KSD, while age and hypertension were not significant factors. After adjusting for gender, BMI, diabetes, and hyperlipidemia in a multivariate logistic regression model, PFT remained an independent and robust predictor of KSD (OR = 1.20, 95% CI 1.15–1.25, *P* < 0.001), while BMI was no longer significant (OR = 1.01, *P* = 0.764) (Fig. 3B). In addition, a slight positive association was noticed between PFT and BMI (Supplementary Material 3).

### Diagnostic performance of PFT and BMI in patients with KSD

ROC curve analysis was utilized to compare the diagnostic abilities of BMI and PFT in predicting KSD. BMI had a moderate ability to discriminate, with AUC values of 0.678 (95% CI: 0.485–0.809) in males and 0.636 (95% CI: 0.441–0.847) in females (Fig. 3C, E). However, PFT had a much better diagnostic performance. In males, it reached AUCs of 0.804 (95% CI: 0.730–0.801), and in females, the AUCs were 0.855 (95% CI: 0.695–0.898) (Fig. 3D, F), which indicated that it was better than BMI in clinical screening for KSD.

### The identification of OB-related differentially expressed genes (ORDEGs)

**Fig. 4:**
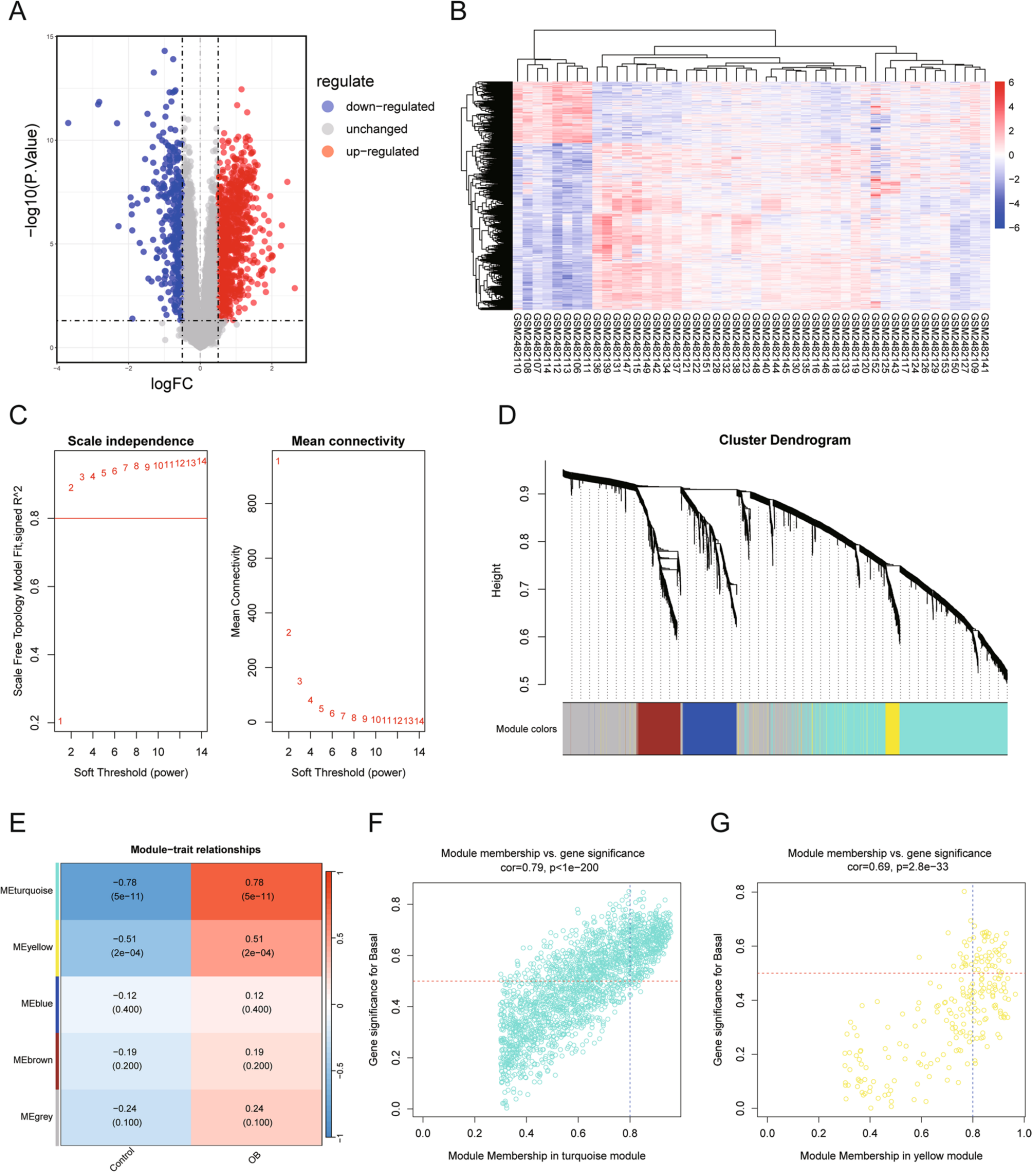
Identification of OB-associated DEGs and OB-related gene modules via WGCNA. **A** Volcano plot and (**B**) heatmap of OB-associated DEGs between the OB and control groups in the GSE94752 dataset. **C** Scale independence and mean connectivity analyses for determining the optimal soft threshold power (β = 2). **D** Hierarchical clustering dendrogram of co-expressed genes, with modules color-coded. **E** Module‒trait correlations: rows represent gene modules, and columns indicate clinical outcomes. Correlation coefficients (red: positive, green: negative) and *P* values (in parentheses) are shown. (F, G) Scatter plots of module membership versus gene significance for KSD in the turquoise **F** and yellow **G** modules. Genes meeting the threshold (module membership > 0.8, gene significance > 0.5) were prioritized as key module genes. OB: obesity; DEGs: differentially expressed genes; WGCNA: weighted gene co-expression networks

**Fig. 5:**
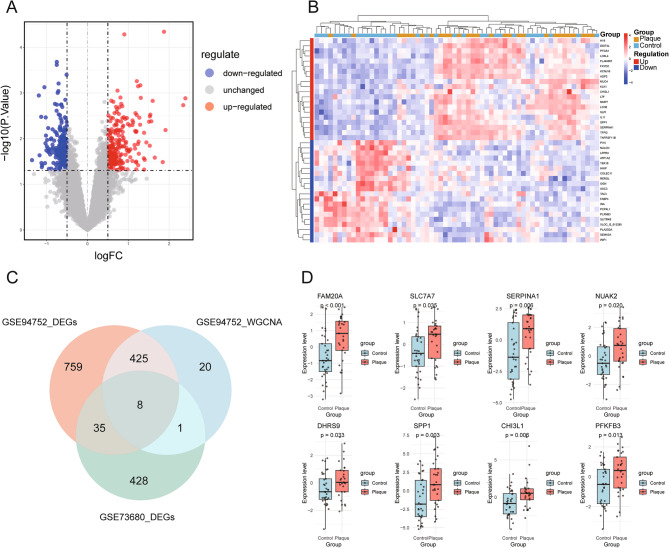
Identification of KSD-associated DEGs and OB-related DEGs (ORDEGs). **A** Volcano plot and (**B**) heatmap of KSD-associated DEGs between the KSD and control groups in the GSE73680 dataset. **C** Venn diagram showing the overlap among OB-associated DEGs, KSD-associated DEGs, and key module genes. The intersecting genes were identified as ORDEGs. **D** Box plot illustrating the expression patterns of the eight candidate ORDEGs. DEGs: differentially expressed genes; KSD: kidney stone disease; OB: obesity; ORDEGs: obesity-related differentially expressed genes

The GSE94752 dataset was subject to transcriptomic analysis for exploring the molecular basis of OB and its connection to KSD. 1,227 DEGs were identified, among which 896 were upregulated and 331 were downregulated in the OB group (Fig. 4A, B; Supplementary Material 4). WGCNA generated a scale-free co-expression network (Fig. 4C) comprising five modules (Fig. 4D). The turquoise and yellow modules displayed the strongest associations with OB (Fig. 4E). Filtering for genes with module membership > 0.8 and gene significance > 0.5 identified 454 hub transcripts—412 within turquoise (Fig. 4F) and 42 within yellow (Fig. 4G; Supplementary Material 5). The analysis of DEGs in the GSE73680 dataset (comparing the plaque group with the control group) disclosed 472 DEGs, with 254 up-regulated and 218 down-regulated genes (Fig. 5A, B; Supplementary Material 6). Eight genes, namely FAM20A, SLC7A7, SERPINA1, DHRS9, SPP1, and CHI3L1, were identified as ORDEGs through the intersection of ORDEGs, key module genes, and KSD-associated DEGs (Fig. 5C), and their expression levels were presented in box plots (Fig. 5D).

### Identification of hub ORDEGs via machine learning

**Fig. 6:**
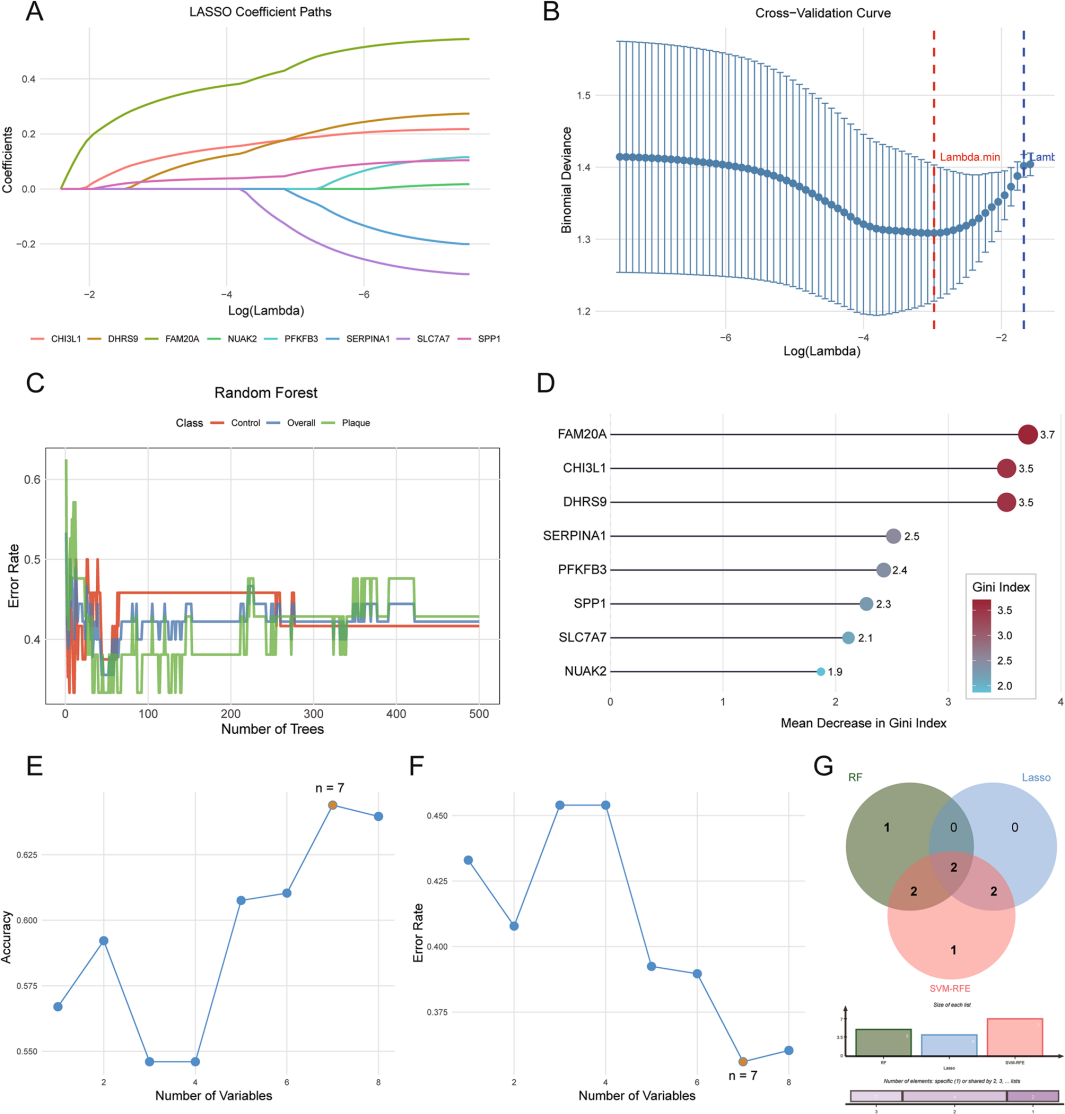
Identification of hub ORDEGs associated with KSD via machine learning approaches. **A**, **B** LASSO regression analysis of the eight candidate ORDEGs. **C** RF classification tree. **D** Variable importance ranking based on the Gini index from the RF algorithm. **E**, **F** Optimal feature selection of ORDEGs via the SVM-RFE algorithm. **G** Venn diagram showing consensus genes from three methods: the top 5 genes from random forest, the 4 genes from LASSO regression, and the 7 feature genes from SVM-RFE analysis. KSD: Kidney stone disease; ORDEGs: obesity-related differentially expressed genes; LASSO: Least absolute shrinkage and selection operator; RF: Random Forest; SVM-RFE: Support vector machine-based feature selection technique

Three machine-learning algorithms were utilized to find out the crucial biomarkers. Four ORDEGs, namely FAM20A, DHRS9, SPP1, and CHI3L1, were identified by LASSO regression (Fig. 6A, B). The RF model, according to Gini importance, ranked eight genes related to the objective (Fig. 6C, D). The SVM-RFE analysis picked out the top seven genes with the best classification performance (Fig. 6E, F). The combination of all three models led to the identification of FAM20A and DHRS9 as the most stable hub genes (Fig. 6G).

### The construction of the OB-related KSD prediction model

**Fig. 7:**
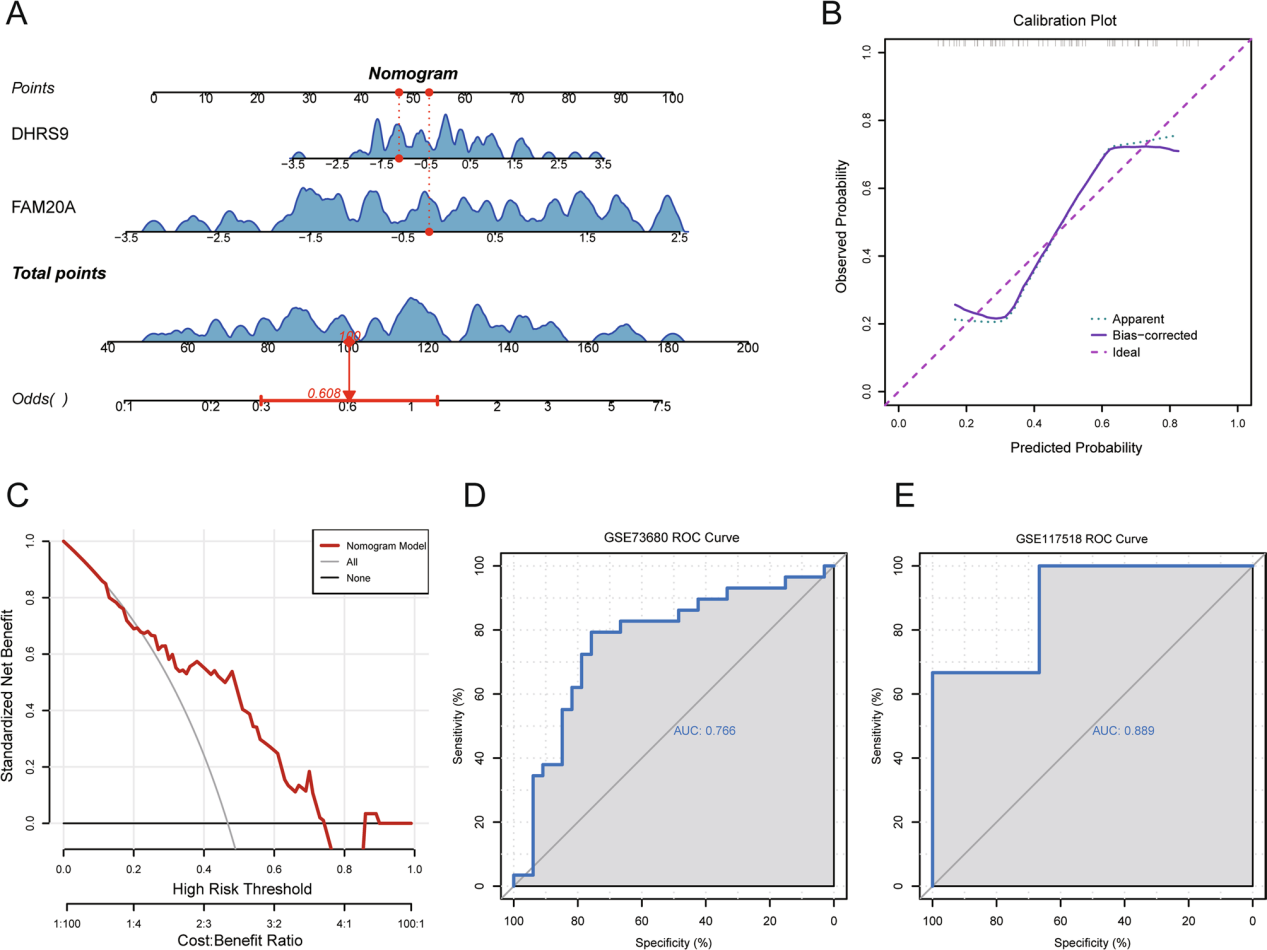
Development and validation of the diagnostic nomogram model for KSD. **A** Nomogram constructed on the basis of the two hub genes. **B** Calibration curve assessing the agreement between the predicted and observed KSD probabilities. **C** DCA curve evaluating the clinical utility of the nomogram. **D**-**E** ROC curves demonstrating the model’s diagnostic performance in the (**D**) internal GSE73680 dataset and (**E**) external validation GSE117518 dataset from GEO. KSD: Kidney stone disease; DCA: decision curve analysis; ROC: operating characteristic curves

A nomogram was built by means of logistic regression which included FAM20A and DHRS9 (Fig. 7A) for the purpose of improving the diagnostic accuracy of OB-related KSD. The calibration curves presented a high level of accordance between the predicted probabilities and the actual ones (Fig. 7B). The decision curve analysis indicated a net clinical benefit across threshold probabilities in the range of 10–80% (Fig. 7C). The model performed well in the internal GSE73680 dataset (AUC = 76.6%; Fig. 7D) and achieved excellent generalizability in the external validation dataset GSE117518 (AUC = 88.9%; Fig. 7E), affirming its diagnostic utility.

### Enrichment of the pathway for the two hub genes

**Fig. 8:**
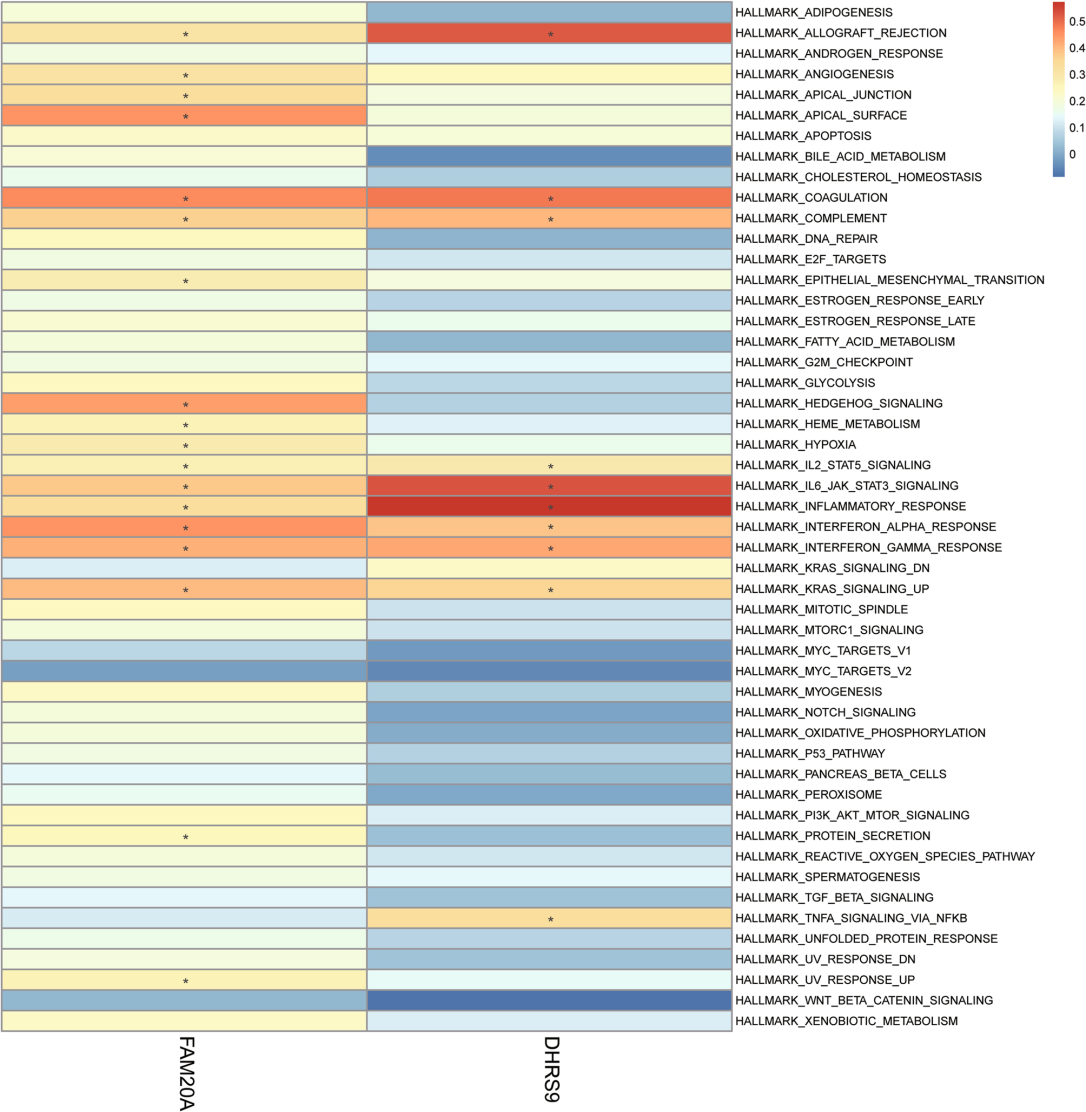
Pathway enrichment of the two hub genes. Pathway enrichment analysis of hub genes (FAM20A/DHRS9), showing significant correlation with inflammatory and metabolic pathways (e.g., IL-6/JAK/STAT3, TNF-α/NF-κB). *P < 0.05, **P < 0.01, ***P < 0.001, ns, *P* ≥ 0.05

Analysis of the pathway enrichment of the two hub genes showed that they were both involved in important inflammatory and immune-related pathways. A positive correlation was found between FAM20A and DHRS9 and certain pathways such as those related to IL-6/JAK/STAT3 signaling, inflammatory response, interferon alpha response, and interferon gamma response. Moreover, FAM20A had a positive connection with protein secretion, and DHRS9 was positively related to TNFA signaling through NF-κB (Fig. 8; Supplementary Material 7). These findings indicate both common and different biological functions in the development of OB-related KSD.

## Discussion

The incidence and recurrence rates of KSD are on the rise globally, which has become an increasingly concerning issue for global health, and it has wide-ranging impacts on the quality of life and healthcare systems all over the world [28, 29]. OB has emerged as a key contributor to the rising burden of KSD [30], with increasing evidence highlighting the role of adipose tissue, particularly perirenal fat as a pathogenic factor [29]. Unlike traditional OB indices such as BMI, perirenal fat more precisely reflects visceral adiposity and its associated metabolic disturbances [5, 31]. This study showed that PFT, a practical and clinically available imaging biomarker, is a strong predictor of KSD risk, and it is better than BMI in both diagnostic value and clinical use.

Previous research [9, 18, 31] has indicated an association between OB and KSD, and this was further confirmed by the present findings. However, while BMI has long been used to define OB, it remains a blunt instrument for evaluating metabolic risk due to its inability to distinguish visceral from subcutaneous fat [12]. The relationship between BMI and KSD has remained inconsistent across studies: while some reported no association [5], others such as Semins et al. [32] noted increased KSD risk with BMI > 30 kg/m². In contrast, our multivariate regression analysis identified PFT (OR = 1.20, *P* < 0.001) as an independent predictor of KSD, whereas BMI was non-significant (OR = 1.01, *P* = 0.764), reinforcing the inadequacy of BMI for stratifying KSD risk. The ROC curve analysis further verified that PFT has a better diagnostic performance compared to BMI, highlighting the potential of PFT as a more accurate clinical tool for evaluating the risk of KSD.

Recent studies further support the diagnostic use of PFT. Increased perirenal fat volume (PFV) was observed in kidneys affected by calculi, as evidenced by Lama et al. [18] and Tastemur et al. [33]. It’s suggested that PFV > 387 cm³ independently predicts KSD and outperforms BMI [33]. Nevertheless, the measurement of perirenal fat volume demands advanced 3D reconstruction software and is not practical in many routine scenarios. In contrast, PFT can be rapidly measured via standard CT or even ultrasound, and it has a high correlation with PFV [34], which makes it a more practical alternative. Consequently, PFT strikes an excellent balance between its ability to diagnose and its practicality in a clinical setting, particularly in hectic healthcare settings.

In addition to clinical findings, the study utilized transcriptomic and machine-learning methods to disclose the molecular mechanisms between perirenal fat and KSD. Two hub genes, namely FAM20A and DHRS9, were singled out by means of integrated LASSO, RF, and SVM-RFE analysis with the utilization of RNA-seq data from the GEO database. The fact that they are upregulated in KSD patients and possess a strong diagnostic performance (AUC: 76.6% internal, 88.9% external) highlights their potential as biomarkers. Analysis of pathway enrichment showed that both genes had a significant association with pathways related to inflammation, especially the IL-6/JAK/STAT3 and TNF-α/NF-κB signaling.

It has been known that these pathways are mediators of chronic inflammation related to OB. The short-chain dehydrogenase/reductase family has a multifunctional member named DHRS9. It is involved in oxylipin metabolism and immune regulation, and may play a part in vascular inflammation and atherosclerosis as well [35, 36]. FAM20A, a secreted pseudo kinase essential for biomineralization, regulates calcium-phosphate metabolism and is known to be upregulated in conditions such as ST-elevation myocardial infarction, also via the IL-6/JAK/STAT3 signaling [37]. Research has demonstrated that perirenal fat releases inflammatory cytokines, including IL-6 and TNF-α [13, 38, 39]. The expression of FAM20A and DHRS9 may be modulated in turn by these cytokines, which might play a role in mediating the crosstalk between adipose and renal in stone formation. In fact, it has been demonstrated that DHRS9 is co-regulated with JAK-STAT components during selenium-mediated hepatoprotection [40], which indicates its participation in inflammatory signaling. All in all, these results suggest a reasonable mechanistic theory: cytokines originating from perirenal fat can activate inflammatory routes (IL-6/JAK/STAT3 and TNF-α/NF-κB), which can cause an upregulation of FAM20A and DHRS9, thus leading to local renal inflammation and the formation of stones. Although this hypothesis seems persuasive, it still needs more experimental verification by means of animal models and in vitro assays.

### Strengths and limitations

There were several remarkable advantages in this study. First, by using routine CT imaging without depending on complex 3D quantification, it validated PFT as a practical, imaging-based biomarker for KSD risk, thereby offering a scalable tool for front-line clinicians. Second, by means of integrating multi-cohort transcriptomic data with machine learning algorithms such as LASSO, RF and SVM-RFE, the identified biomarkers can have their robustness enhanced. Third, a link between PFT and particular inflammatory pathways were established in our study. This promoted the understanding of the mechanism of adipose-induced stone formation and provided a basis for precision medicine methods.

Nevertheless, a number of limitations should be recognized. Key lifestyle and metabolic confounders’ data were absent in this study, including dietary patterns, physical activity, serum uric acid and calcium levels, and the use of medications like diuretics. These factors may have an impact on the risk of stone formation. Furthermore, although bioinformatics and MR techniques offered strong evidence of association, no experimental confirmations (for example, in animal models or cellular assays) were carried out to verify causality in the adipose-inflammation-stone route. Finally, even though PFT can be measured with CT or ultrasound, the availability of imaging modalities varies among different healthcare settings, especially in regions with limited resources. This highlights the necessity of validating sonographic PFT in the future as a more widely applicable tool.

## Conclusion

In this research, PFT was found to be a useful and better predictor for KSD compared to BMI in terms of clinical risk evaluation. Bioinformatics and machine learning revealed that FAM20A and DHRS9 became important biomarkers associated with inflammatory pathways, specifically IL-6/JAK/STAT3 and TNF-α/NF-κB, indicating a connection in the mechanism between perirenal fat and KSD. These results provided a basis for prevention and management strategies targeted at OB-related KSD.

## Supplementary Information

Supplementary Material 1.

Supplementary Material 2.

Supplementary Material 3.

Supplementary Material 4.

Supplementary Material 5.

Supplementary Material 6.

Supplementary Material 7.

Supplementary Material 8.

## Data Availability

The GSE94752, GSE73680 and GSE117518 datasets supporting the conclusions of this study are available in the GEO database (https://www.ncbi.nlm.nih.gov/geo/). The hallmark gene sets (h.all.v2024.1.Hs.symbols.gmt) were obtained from the Molecular Signatures Database (MSigDB) (https://www.gsea-msigdb.org/gsea/msigdb). The GWAS data for obesity (exposure) and nephrolithiasis (outcome) were obtained from the IEU GWAS database (https://gwas.mrcieu.ac.uk/). The competed tomography (CT) imaging data was available from the corresponding author and can be available upon reasonable request.

## Abbreviations

PFT: Perirenal fat thickness
KSD: Kidney stone disease
PF: Perirenal fat
OB: Obesity
BMI: Body mass index
GEO: Gene Expression Omnibus
WGCNA: Weighted Gene Co-expression Network Analysis
TOM: Topological overlap matrix
MM: Module membership
GS: Gene significance
DEGs: Differentially expressed genes
ORDEGs: OB-related differentially expressed genes
RF: Random forest
LASSO: Least absolute shrinkage and selection operator
SVM-RFE: Support vector machine-based feature selection technique
ROC: Receiver operating characteristic
AUC: Area under the curve
OR: Odds ratio
PFV: Perirenal fat volume
RPT: Renal parenchymal thickness
STEMI: ST-segment elevation myocardial infarction
