# Supplementary material for: A cross-sectional and bioinformatics-based analysis: perirenal fat thickness as a superior predictor of kidney stone disease
Source: Lipids Health Dis. 2025 Aug 29;24:269. doi: 10.1186/s12944-025-02686-4 (PMC12395729; doi:10.1186/s12944-025-02686-4)
Supplement: Supplementary file 1 — Supplementary Material 1. [file 12944_2025_2686_MOESM1_ESM.docx]

**Supplementary Material 1. Mendelian randomization (MR) analysis methods**

A two-sample MR approach was utilized to explore the causal link between obesity and nephrolithiasis (19). Summary-level Genome-Wide Association Studies (GWAS) data for obesity (exposure) and nephrolithiasis (outcome) were obtained from the IEU GWAS database (<https://gwas.mrcieu.ac.uk/>) with IDs finn-b-E4_OBESITY and ebi-a-GCST90018715, respectively. The obesity dataset comprised 218,735 European individuals (8,908 cases and 209,827 controls), while the nephrolithiasis dataset included 178,726 East Asian individuals (11,699 cases and 167,027 controls). The MR methodology necessitates adherence to three fundamental assumptions: (1) selected genetic variants (SNPs) must strongly associate with obesity (*P* < 5 × 10^-7^); (2) these SNPs must be independent of confounding factors; and (3) SNPs should influence nephrolithiasis solely through obesity. The causal effect of obesity on nephrolithiasis was estimated using inverse-variance weighted (IVW) meta-analysis of Wald ratios. MR-Egger regression assessed horizontal pleiotropy (intercept *P* > 0.05 indicating absence), and leave-one-out sensitivity analyses evaluated robustness. Statistical significance was defined as *P* < 0.05 (two-tailed). Analyses were performed using MR-specific tools, including "two-sample-MR", "MR-PRESSO" and "mr.raps".
