# Supplementary material for: A cross-sectional and bioinformatics-based analysis: perirenal fat thickness as a superior predictor of kidney stone disease
Source: Lipids Health Dis. 2025 Aug 29;24:269. doi: 10.1186/s12944-025-02686-4 (PMC12395729; doi:10.1186/s12944-025-02686-4)
Supplement: Supplementary file 2 — Supplementary Material 2. [file 12944_2025_2686_MOESM2_ESM.docx]

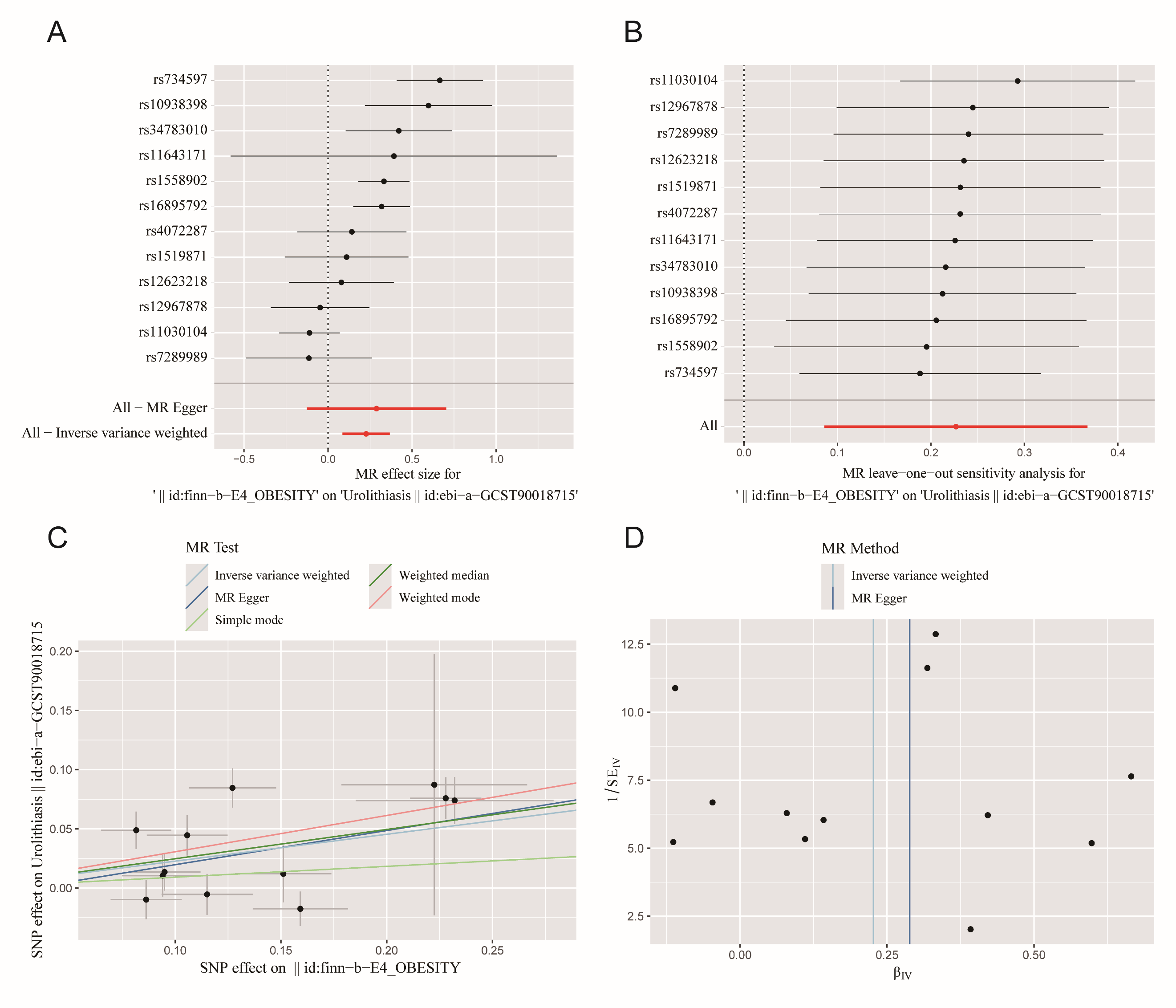


**Supplementary Material 2**. Causal link between OB and KSD risk via Mendelian randomization (MR) analysis. (A) IVW analysis of all SNPs (red points) shows OB-KSD causal linkages (horizontal lines: 95% CIs). (B) Leave-one-out sensitivity analysis (black points) confirms robust causal estimates (red points: full IVW estimates). (C) MR scatterplot revealing consistent effect directions across methods (slopes = effect magnitudes). (D) Funnel plot (vertical lines) visualizing SNP-specific causal estimates.
