# Supplementary material for: A cross-sectional and bioinformatics-based analysis: perirenal fat thickness as a superior predictor of kidney stone disease
Source: Lipids Health Dis. 2025 Aug 29;24:269. doi: 10.1186/s12944-025-02686-4 (PMC12395729; doi:10.1186/s12944-025-02686-4)
Supplement: Supplementary file 3 — Supplementary Material 3. [file 12944_2025_2686_MOESM3_ESM.docx]

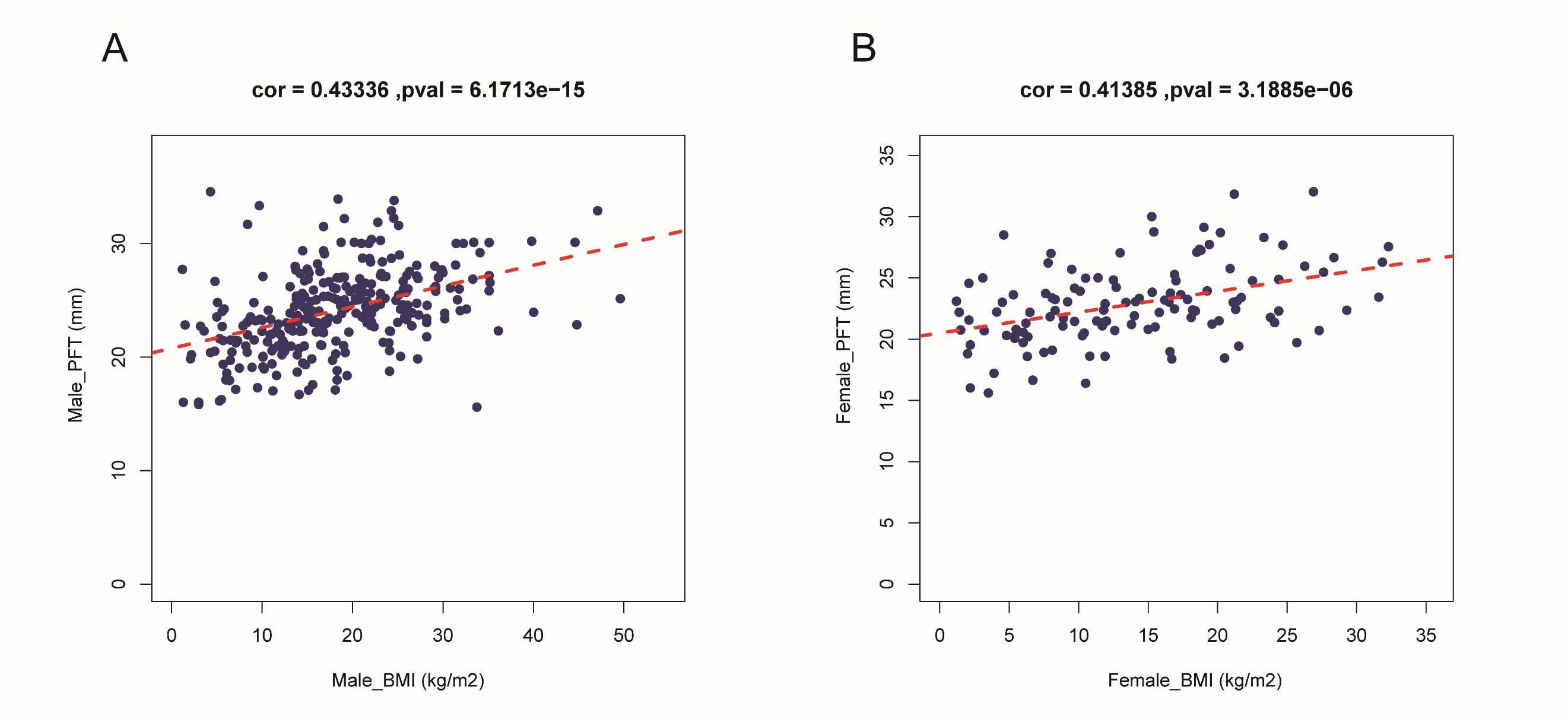


**Supplementary Material 3.** Correlation analysis between PFT results and BMI in males (A) and females (B).
