# Supplementary material for: A cross-sectional and bioinformatics-based analysis: perirenal fat thickness as a superior predictor of kidney stone disease
Source: Lipids Health Dis. 2025 Aug 29;24:269. doi: 10.1186/s12944-025-02686-4 (PMC12395729; doi:10.1186/s12944-025-02686-4)
Supplement: Supplementary file 8 — Supplementary Material 8. [file 12944_2025_2686_MOESM8_ESM.pdf]

none none

A Cross-Sectional and Bioinformatics-Based Analysis: Perirenal Fat Thickness as a Superior Predictor

Vishwakarma Group of Institutions

Document Details

Submission ID

trn:oid:::3117:475921701

Submission Date

Jul 24, 2025, 5:18 PM GMT+8

Download Date

Jul 24, 2025, 5:20 PM GMT+8

File Name

257492099224641031O907_A Cross-Sectional and Bioinformatics-Based Analysis: Perirenal Fat T....docx

File Size

238.6 KB

22 Pages

5,636 Words

31,185 Characters

15% Overall Similarity

The combined total of all matches, including overlapping sources, for each database.

Filtered from the Report

- Bibliography

Match Groups

60 Not Cited or Quoted 15%
Matches with neither in-text citation nor quotation marks

2 Missing Quotations 0%
Matches that are still very similar to source material

0 Missing Citation 0%
Matches that have quotation marks, but no in-text citation

0 Cited and Quoted 0%
Matches with in-text citation present, but no quotation marks

Top Sources

13% Internet sources
11% Publications
0% Submitted works (Student Papers)

Integrity Flags

0 Integrity Flags for Review

No suspicious text manipulations found.

Our system's algorithms look deeply at a document for any inconsistencies that would set it apart from a normal submission. If we notice something strange, we flag it for you to review.

A Flag is not necessarily an indicator of a problem. However, we'd recommend you focus your attention there for further review.

Match Groups

- **60** Not Cited or Quoted 15%
  Matches with neither in-text citation nor quotation marks
- **2** Missing Quotations 0%
  Matches that are still very similar to source material
- **0** Missing Citation 0%
  Matches that have quotation marks, but no in-text citation
- **0** Cited and Quoted 0%
  Matches with in-text citation present, but no quotation marks

Top Sources

13%	Internet sources
11%	Publications
0%	Submitted works (Student Papers)

Top Sources

The sources with the highest number of matches within the submission. Overlapping sources will not be displayed.

#	Type	Source	%
1	Internet	www.frontiersin.org	4%
2	Internet	biosignaling.biomedcentral.com	1%
3	Internet	www.researchsquare.com	<1%
4	Internet	www.mdpi.com	<1%
5	Internet	www.medrxiv.org	<1%
6	Internet	translational-medicine.biomedcentral.com	<1%
7	Internet	discovery.researcher.life	<1%
8	Internet	www.dovepress.com	<1%
9	Internet	etj.bioscientifica.com	<1%
10	Publication	Chang-Qing Liu, Zhong-Bei Yu, Jin-Xian Gan, Tian-Ming Mei. "Preoperative blood ...	<1%

11 Publication

David J. Stensel, Adrianne E. Hardman, Jason M.R. Gill. "Physical Activity and Healt... <1%

12 Publication

Prachi Bajpai, Ravi Paluri, Sameer Al Diffalha, Darshan S Chandrashekar et al. "Dif... <1%

13 Publication

Shokoufeh Khanzadeh, Fatemeh Zarimeidani, Erfan Kashani, Saghar Babadi et al. ... <1%

14 Internet

www.spandidos-publications.com <1%

15 Publication

Yiming Lv, Jinhui Hu, Wenqian Zheng, Lina Shan, Bingjun Bai, Hongbo Zhu, Sheng ... <1%

16 Publication

Chaoqun Hu, Mei Ge, Yan Liu, Wei Tan, Yingzhi Zhang, Min Zou, Lingya Xiang, Xia... <1%

17 Internet

www.biorxiv.org <1%

18 Internet

ec.bioscientifica.com <1%

19 Publication

"Full Issue PDF", Journal of the American College of Cardiology, 2025 <1%

20 Publication

Jong Wook Choi, Chul-min Lee, Bo-Kyeong Kang, Mimi Kim. "Perirenal fat thicknes... <1%

21 Internet

bmcmedicine.biomedcentral.com <1%

22 Internet

feyz.kaums.ac.ir <1%

23 Internet

ntnuopen.ntnu.no <1%

24 Internet

www.nature.com <1%

25	Internet	
journals.lww.com		<1%
26	Publication	
Dekai Hu, Guoxiang Li, Defeng Ge, Leilei Ke, Hongmin Shu, Yang Chen, Zongyao H...		<1%
27	Publication	
Liang Wang, Yilan Sun, Qing Sang, Zheng Wang, Chengyuan Yu, Zhehong Li, Ming...		<1%
28	Publication	
Melissa Premaor, Richard A Parker, Steve Cummings, Kris Ensrud, Jane A Cauley, ...		<1%
29	Publication	
RenJie Jiang, LinLin Ruan, Taohui Ding, Hongtao Wan et al. "Development of a pro...		<1%
30	Publication	
Thomas Hughes, Amelia Pietropaolo, Matthew Archer, Tania Davis, Loretta Tear, ...		<1%
31	Internet	
bant.org.uk		<1%
32	Internet	
d2v96fxpocvxx.cloudfront.net		<1%
33	Internet	
mdpi-res.com		<1%
34	Publication	
Ahmed Saad, Allan B. Dietz, Sandra M.S. Herrmann, LaTonya J. Hickson et al. "Aut...		<1%
35	Publication	
Basavaraj Vastrad, Chanabasayya Vastrad. "Screening and identification of key bi...		<1%
36	Publication	
Haichao Huang, Shi Chen, Wenzhao Zhang, Tao Wang, Peide Bai, Jinchun Xing, Hu...		<1%
37	Publication	
James M. Rippe, John P. Foreyt. "Obesity Prevention and Treatment - A Practical G...		<1%
38	Internet	
doaj.org		<1%

A Cross-Sectional and Bioinformatics-Based Analysis: Perirenal Fat Thickness as a Superior Predictor of Kidney Stone Disease

Abstract

Background: Kidney stone disease (KSD) is a growing global health concern, with obesity (OB) as a major risk factor linked to metabolic dysfunction and chronic inflammation. Although the common method for evaluating obesity is body mass index (BMI), it is not specific enough when it comes to reflecting visceral fat. The perirenal fat thickness (PFT) might present better predictive capabilities. The goal of this research was to assess the clinical usefulness of PFT in the diagnosis of KSD and to clarify the molecular mechanisms connecting OB to KSD.

Methods: Analysis was carried out on a retrospective cohort of 413 patients (265 having KSD and 148 controls). Abdominal computed tomography was used to measure PFT. Three machine-learning methods, weighted gene co-expression network analysis, and differential expression analysis were used to evaluate gene expression data for key gene identification. Internal and external datasets were used to develop and validate a diagnostic nomogram. Also, pathway enrichment analysis was carried out.

Results: KSD patients exhibited greater PFT versus controls, with significantly enhanced diagnostic accuracy compared to BMI. Multivariate analysis confirmed PFT as an independent predictor of KSD (OR = 1.20, $P < 0.001$). Eight genes that are differentially expressed in relation to OB were identified, among which FAM20A and DHRS9 were found to be central hub genes. The nomogram exhibited a high level of

predictive accuracy. Analysis of enrichment pointed to the IL-6/JAK/STAT3 and TNF-α/NF-κB signaling pathways in the connection between perirenal fat and KSD.

Conclusions: PFT serves as a practical and dependable marker for the risk of KSD. It is superior to BMI and can be conveniently incorporated into routine clinical practice. Stone formation may be linked to perirenal fat by FAM20A and DHRS9 via inflammatory pathways, which provides potential targets for the management of OB-related KSD.

Keywords: Kidney stone, Obesity, Body mass index, Perirenal fat thickness, Inflammation

Introduction

The formation of crystalline deposits within the renal system characterizes kidney stone disease (KSD), which is a common urological condition [1, 2]. It brings about a great global health problem and also causes a large amount of economic and healthcare burdens [3, 4]. In China, the rate of a certain renal system-related disease is notably higher than the estimated global prevalence range [5].

Globally, obesity (OB) has become a crucial health problem at the same time. A substantial global adult population was classified as obese in 2015, with projections suggesting that nearly half of the adult population in the United States will be obese (BMI $\geq$ 30) by 2030 [6, 7]. Excessive body weight is only one aspect of OB; it is also closely tied to metabolic dysfunctions like insulin resistance, dyslipidemia, and chronic inflammation [8]. A large number of studies have constantly shown that OB, especially

when combined with metabolic dysfunction and chronic inflammation, greatly elevates the risk of KSD [8-11].

Although body mass index (BMI) is still the traditional method for evaluating OB, it has been criticized because it can't distinguish between visceral and subcutaneous adiposity [12]. Perirenal fat, which is next to the kidneys and is a metabolically active adipose depot that secretes adipokines and pro-inflammatory cytokines [13, 14], is of special interest. Some recent research indicates that the thickness of perirenal fat (PFT) could be a more precise predictor of metabolic disorders related to OB and their complications compared to BMI [15-17].

While existing literature indicates a connection between perirenal fat and KSD, a great deal of the current research depends on advanced 3D imaging technologies and intricate analytical methods, which restricts their use in routine clinical work [5, 18]. On the contrary, standard computed tomography (CT) imaging, which is often used in clinical situations, can be used to measure PFT easily and dependably. This makes it a useful and effective measure for incorporating into regular diagnostic procedures. For clinicians with a busy schedule, PFT is a convenient and time-saving substitute for assessing KSD risk in obese patients without requiring specialized devices.

36

This research measured the association between PFT and KSD risk. In addition, it aimed to find out possible molecular biomarkers and clarified the mechanistic paths connecting the fat around the kidney to stone formation, thus offering understanding for clinical decision-making and personalized risk evaluation strategies.

Methods

The design of the study and the patient group

Formal approval for this research was granted by the Ethics Committee of the University of Hong Kong - Shenzhen Hospital (Approval No. [2025]041). In a single institute, a retrospective analysis was carried out on 840 patients who were admitted to the Urology Department between January and December in 2020. Based on kidney stone status, participants were categorized into two groups: KSD group (n = 265; unilateral kidney stone-positive) and control group (n = 148; bilateral kidney stone-negative).

The exclusion criteria included urologic tumors (n = 28), renal atrophy (n = 13), moderate-to-severe hydronephrosis (n = 23), ureteral strictures (n = 20), unilateral renal agenesis (n = 28), renal cysts (n = 15), and bilateral kidney stones (n = 300). Figure 1 schematizes the methodological workflow.

Baseline characteristics included age, gender, BMI, and history of: (1) diabetes mellitus (elevated fasting glucose ≥ 7.0 mmol/L or HbA1c ≥ 6.5%); (2) hypertension (systolic blood pressure [SBP] ≥140 mmHg and/or diastolic blood pressure [DBP] ≥ 90 mmHg on ≥2 separate occasions); and (3) hyperlipidemia (total cholesterol ≥ 5.2 mmol/L and/or LDL-C ≥ 3.4 mmol/L and/or triglycerides ≥1.7 mmol/L).

The collection of computed tomography (CT) image data and the measurement of the perirenal fat thickness (PFT)

Two scanner platforms were used to perform standard abdominal CT scans on all

iThenticate Page 9 of 27 - Integrity Submission Submission ID trn:oid:::3117:475921701

participants in the supine position: (1) The GE Lightspeed Ultra 16 (64 - detector rows, 1.25 mm slice thickness, pitch 1.5, tube voltage 120 kVP, and mAs in the range of 100 - 200 [automated modulation]); (2) The Siemens Somatom Definition AS (1.0 mm slice thickness, pitch 1.2, tube voltage 120 kVP, and mAs 100 - 200). High - resolution algorithms were used by both scanner platforms to generate multiplanar reconstructions (with a thickness of 3 mm and intervals of 3 mm). Axial CT sections were used to quantify PFT as per Mayo Clinic standards [19], with the measurement of the horizontal distance between the renal parenchymal border and the posterior rectus sheath along the renal venous axis (Figure 2). All measurements were carried out by two radiologists who were certified and blinded to all clinical information. For the subjects in the control group, the largest value of either the left or right PFT was chosen for analysis.

The acquisition of microarray data

Microarray datasets associated with OB and KSD were downloaded from the Gene Expression Omnibus (GEO; http://www.ncbi.nlm.nih.gov/geo): GSE94752 (OB discovery cohort), GSE73680 (KSD discovery cohort), and GSE117518 (KSD validation cohort). These data formed the basis for subsequent transcriptomic and machine-learning analyses.

Co-expression gene modules analysis

To identify co-expressed gene modules and evaluate their correlations with clinical traits, the weighted gene co-expression network analysis (WGCNA) method was

utilized [20, 21]. A subset of highly variable genes was selected for network construction. A soft-thresholding power of $\beta = 2$ was selected to satisfy scale-free topology criteria (scale-free $R^2 > 0.8$). A signed topological overlap matrix (TOM) was then calculated, followed by dynamic tree-cutting (minimum module size = 100 genes, merge cut height = 0.25), yielding five discrete modules. Module eigengenes were correlated with obesity status using Pearson's test; modules surpassing $|r| > 0.5$ and FDR < 0.05 were considered trait-relevant. Within these modules, genes exhibiting module membership (MM) > 0.8 and gene significance (GS) > 0.5 were retained as hub candidates for subsequent integration.

The identification of genes with differential expression (DEGs)

Differentially expressed genes (DEGs) were identified by using the limma package (v4.4.3) in R. An empirical Bayes moderation-incorporated linear model was applied. Genes with $P < 0.05$ and absolute log fold change > 0.5 were considered statistically significant. Venn diagrams were used to visualize the genes obtained from the intersecting of DEGs lists of GSE94752 and GSE73680 and the key WGCNA modules to identify OB-related DEGs (ORDEGs). Volcano plots in ggplot2 and heatmaps in pheatmap were created for visualizations.

Algorithms in machine learning

Hub genes related to diseases can be effectively identified by machine-learning methods. For biomarker screening, several well-known machine learning algorithms

are widely used, like random forest (RF), least absolute shrinkage and selection operator (LASSO), and support vector machine-based feature selection technique (SVM-RFE) as mentioned in the previous research [22]. Variable filtration is carried out by LASSO regression to reduce overfitting [23]. When it comes to handling high-dimensional datasets, random forest (RF) has an advantage, which allows for strong predictive modeling and precise estimation of variable importance [24]. The SVM-RFE method was used to gradually remove the least significant variables, and finally optimize the subset of crucial genes for better classification performance [22]. The hub ORDEGs in the KSD were determined as the overlapping genes found by these three machine learning algorithms, and they were shown in a Venn diagram.

The construction and validation of nomogram

Using the rms package in R [25], diagnostic nomograms for OB-related KSD were built. There were two main scales in the nomogram: one was a "points" scale, which gave individual scores to each gene, and the other was a "total points" scale, indicating the cumulative contribution of all genes. Calibration curves and decision curve analysis (DCA) were utilized to appraise the clinical prediction efficiency of the nomogram. Also, the discriminative capacity of the nomogram was evaluated by means of receiver operating characteristic (ROC) curve analysis. A value of area under the curve (AUC) more than 0.7 was considered clinically significant, which showed strong predictive power [26]. The generalizability of the nomogram was evaluated by using the GSE117518 dataset for external validation.

Analysis of pathway enrichment

Single-sample gene set enrichment analysis (ssGSEA) was performed using the GSVA package (v1.48.3) and MSigDB hallmark gene sets (h.all.v2024.1.Hs.symbols.gmt) [27]. Significant biomarker-pathway correlations (Pearson, $P < 0.05$) were identified via heatmap visualization. Scatter plots and regression analysis further highlighting the top associations.

Statistical analysis

R software (version 4.3.2), GraphPad Prism (version 8.0.2), and SPSS (version 25.0) were utilized for statistical analyses. When it comes to continuous variables, Student's t test was used to make comparisons between two groups. Either the chi-square (χ^2) test or Fisher's exact test, depending on the data distribution, was used to analyze categorical variables. Pearson's correlation coefficient was utilized to analyze the relationship between PFT results and BMI. To assess the clinical predictors of KSD, univariate and multivariate regression analyses were utilized. A two-tailed $P < 0.05$ was considered statistically significant.

Results

Clinical characteristics at the baseline

A total of 265 patients with KSD and 148 controls were included in this study. Both groups had comparable mean ages (54.4 vs. 53.6 years, $P = 0.646$). However, a significantly higher proportion of males was observed in the KSD group compared to

controls (77.7% vs. 60.1%, P = 0.029), and patients with KSD exhibited a notably higher BMI (24.48 vs. 22.45 kg/m2, P < 0.001). Importantly, PFT was significantly elevated in the KSD group (20.35 vs. 10.98 mm, P < 0.001), reinforcing its potential association with stone formation. Additionally, the prevalence of diabetes (24.9% vs. 16.2%, P = 0.040) and hyperlipidemia (23.8% vs. 10.8%, P = 0.001) was significantly higher in the KSD group. However, no significant difference was found in hypertension prevalence (34.0% vs. 27.7%, P = 0.19). A summary of these clinical characteristics can be found in Table 2.

The causal link between OB and KSD.

Mendelian randomization (MR) analysis was carried out first (Supplementary Materials 1 and 2) in order to evaluate whether OB has a causal effect on the risk of KSD. The findings of the MR analysis hinted at a possible causal connection between OB and the emergence of KSD. Clinical data were used to further investigate this association. Figure 3A showed that in univariate logistic regression, gender, BMI, PFT, diabetes, and hyperlipidemia were identified as important risk factors for KSD, while age and hypertension were not significant factors. After adjusting for gender, BMI, diabetes, and hyperlipidemia in a multivariate logistic regression model, PFT remained an independent and robust predictor of KSD (OR = 1.20, 95% CI 1.15–1.25, P < 0.001), while BMI was no longer significant (OR = 1.01, P = 0.764) (Figure 3B). In addition, a slight positive association was noticed between PFT and BMI (Supplementary Material 3).

Diagnostic performance of PFT and BMI in patients with KSD

ROC curve analysis was utilized to compare the diagnostic abilities of BMI and PFT in predicting KSD. BMI had a moderate ability to discriminate, with AUC values of 0.678 (95% CI: 0.485–0.809) in males and 0.636 (95% CI: 0.441–0.847) in females (Figure 3C, E). However, PFT had a much better diagnostic performance. In males, it reached AUCs of 0.804 (95% CI: 0.730–0.801), and in females, the AUCs were 0.855 (95% CI: 0.695–0.898) (Figure 3D, F), which indicated that it was better than BMI in clinical screening for KSD.

The identification of OB-related differentially expressed genes (ORDEGs)

The GSE94752 dataset was subject to transcriptomic analysis for exploring the molecular basis of OB and its connection to KSD. 1,227 DEGs were identified, among which 896 were upregulated and 331 were downregulated in the OB group (Figure 4A, B; Supplementary Material 4). WGCNA generated a scale-free co-expression network (Figure 4C) comprising five modules (Figure 4D). The turquoise and yellow modules displayed the strongest associations with obesity (Figure 4E). Filtering for genes with module membership > 0.8 and gene significance > 0.5 identified 454 hub transcripts—412 within turquoise (Figure 4F) and 42 within yellow (Figure 4G; Supplementary Material 5). The analysis of DEGs in the GSE73680 dataset (comparing the plaque group with the control group) disclosed 472 DEGs, with 254 up-regulated and 218 down-regulated genes (Figure 5A, B; Supplementary Material 6). Eight genes, namely FAM20A, SLC7A7, SERPINA1, DHRS9, SPP1, and CHI3L1, were identified as

ORDEGs through the intersection of ORDEGs, key module genes, and KSD-associated DEGs (Figure 5C), and their expression levels were presented in box plots (Figure 5D).

Identification of hub ORDEGs via machine learning

Three machine-learning algorithms were utilized to find out the crucial biomarkers. Four ORDEGs, namely FAM20A, DHRS9, SPP1, and CHI3L1, were identified by LASSO regression (Figure 6A, B). The RF model, according to Gini importance, ranked eight genes related to the objective (Figure 6C, D). The SVM-RFE analysis picked out the top seven genes with the best classification performance (Figure 6E, F). The combination of all three models led to the identification of FAM20A and DHRS9 as the most stable hub genes (Figure 6G).

The construction of the OB-related KSD prediction model

A nomogram was built by means of logistic regression which included FAM20A and DHRS9 (Figure 7A) for the purpose of improving the diagnostic accuracy of OB-related KSD. The calibration curves presented a high level of accordance between the predicted probabilities and the actual ones (Figure 7B). The decision curve analysis indicated a net clinical benefit across threshold probabilities in the range of 10% to 80% (Figure 7C). The model performed well in the internal GSE73680 dataset (AUC = 76.6%; Figure 7D) and achieved excellent generalizability in the external validation dataset GSE117518 (AUC = 88.9%; Figure 7E), affirming its diagnostic utility.

Enrichment of the pathway for the two hub genes

Analysis of the pathway enrichment of the two hub genes showed that they were both involved in important inflammatory and immune-related pathways. A positive correlation was found between FAM20A and DHRS9 and certain pathways such as those related to IL-6/JAK/STAT3 signaling, inflammatory response, interferon alpha response, and interferon gamma response. Moreover, FAM20A had a positive connection with protein secretion, and DHRS9 was positively related to TNFA signaling through NF-κB (Figure 8; Supplementary Material 7). These findings indicate both common and different biological functions in the development of OB-related KSD.

Discussion

The incidence and recurrence rates of KSD are on the rise globally, which has become an increasingly concerning issue for global health, and it has wide - ranging impacts on the quality of life and healthcare systems all over the world [28, 29]. OB has emerged as a key contributor to the rising burden of KSD [30], with increasing evidence highlighting the role of adipose tissue, particularly perirenal fat as a pathogenic factor [29]. Unlike traditional OB indices such as BMI, perirenal fat more precisely reflects visceral adiposity and its associated metabolic disturbances [5, 31]. This study showed that PFT, a practical and clinically available imaging biomarker, is a strong predictor of KSD risk, and it is better than BMI in both diagnostic value and clinical use.

Previous research [9, 18, 31] has indicated an association between OB and KSD, and this was further confirmed by the present findings. However, while BMI has long been

used to define OB, it remains a blunt instrument for evaluating metabolic risk due to its inability to distinguish visceral from subcutaneous fat [12, 32]. The relationship between BMI and KSD has remained inconsistent across studies: while some reported no association [5], others such as Semins et al. [33] noted increased KSD risk with BMI > 30 kg/m^2. In contrast, our multivariate regression analysis identified PFT (OR = 1.20, $P < 0.001$) as an independent predictor of KSD, whereas BMI was non-significant (OR = 1.01, $P = 0.764$), reinforcing the inadequacy of BMI for stratifying KSD risk. The ROC curve analysis further verified that PFT has a better diagnostic performance compared to BMI, highlighting the potential of PFT as a more accurate clinical tool for evaluating the risk of KSD.

Recent studies further support the diagnostic use of PFT. Increased perirenal fat volume (PFV) was observed in kidneys affected by calculi, as evidenced by Lama et al. [18] and Tastemur et al. [34]. It's suggested that PFV > 387 cm^3 independently predicts KSD and outperforms BMI [34]. Nevertheless, the measurement of perirenal fat volume demands advanced 3D reconstruction software and is not practical in many routine scenarios. In contrast, PFT can be rapidly measured via standard CT or even ultrasound, and it has a high correlation with PFV [35], which makes it a more practical alternative. Consequently, PFT strikes an excellent balance between its ability to diagnose and its practicality in a clinical setting, particularly in hectic healthcare settings.

In addition to clinical findings, the study utilized transcriptomic and machine-learning methods to disclose the molecular mechanisms between perirenal fat and KSD. Two hub genes, namely FAM20A and DHRS9, were singled out by means of integrated

LASSO, RF, and SVM-RFE analysis with the utilization of RNA-seq data from the GEO database. The fact that they are upregulated in KSD patients and possess a strong diagnostic performance (AUC: 76.6% internal, 88.9% external) highlights their potential as biomarkers. Analysis of pathway enrichment showed that both genes had a significant association with pathways related to inflammation, especially the IL-6/JAK/STAT3 and TNF-α/NF-κB signaling.

It has been known that these pathways are mediators of chronic inflammation related to OB. The short-chain dehydrogenase / reductase family has a multifunctional member named DHRS9. It is involved in oxylipin metabolism and immune regulation, and may play a part in vascular inflammation and atherosclerosis as well [36, 37]. FAM20A, a secreted pseudo kinase essential for biomineralization, regulates calcium-phosphate metabolism and is known to be upregulated in conditions such as ST-elevation myocardial infarction, also via the IL-6/JAK/STAT3 signaling [38]. Research has demonstrated that perirenal fat releases inflammatory cytokines, including IL-6 and TNF-α [13, 39, 40]. The expression of FAM20A and DHRS9 may be modulated in turn by these cytokines, which might play a role in mediating the crosstalk between adipose and renal in stone formation. In fact, it has been demonstrated that DHRS9 is co-regulated with JAK-STAT components during selenium-mediated hepatoprotection [41], which indicates its participation in inflammatory signaling. All in all, these results suggest a reasonable mechanistic theory: cytokines originating from perirenal fat can activate inflammatory routes (IL-6/JAK/STAT3 and TNF-α/NF-κB), which can cause an upregulation of FAM20A and DHRS9, thus leading to local renal inflammation and

the formation of stones. Although this hypothesis seems persuasive, it still needs more experimental verification by means of animal models and in vitro assays.

Strengths and limitations

There were several remarkable advantages in this study. First, by using routine CT imaging without depending on complex 3D quantification, it validated PFT as a practical, imaging-based biomarker for KSD risk, thereby offering a scalable tool for front-line clinicians. Second, by means of integrating multi-cohort transcriptomic data with machine learning algorithms such as LASSO, RF and SVM-RFE, the identified biomarkers can have their robustness enhanced. Third, a link between PFT and particular inflammatory pathways were established in our study. This promoted the understanding of the mechanism of adipose-induced stone formation and provided a basis for precision medicine methods.

Nevertheless, a number of limitations should be recognized. Key lifestyle and metabolic confounders' data were absent in this study, including dietary patterns, physical activity, serum uric acid and calcium levels, and the use of medications like diuretics. These factors may have an impact on the risk of stone formation. Furthermore, although bioinformatics and MR techniques offered strong evidence of association, no experimental confirmations (for example, in animal models or cellular assays) were carried out to verify causality in the adipose-inflammation-stone route. Finally, even though PFT can be measured with CT or ultrasound, the availability of imaging modalities varies among different healthcare settings, especially in regions with limited resources. This highlights the necessity of validating sonographic PFT in the future as

a more widely applicable tool.

Conclusion

In this research, PFT was found to be a useful and better predictor for KSD compared to BMI in terms of clinical risk evaluation. Bioinformatics and machine learning revealed that FAM20A and DHRS9 became important biomarkers associated with inflammatory pathways, specifically IL-6/JAK/STAT3 and TNF-α/NF-κB, indicating a connection in the mechanism between perirenal fat and KSD. These results provided a basis for prevention and management strategies targeted at OB-related KSD.

References

1. Shastri S, Patel J, Sambandam KK, Lederer ED: **Kidney Stone Pathophysiology, Evaluation and Management: Core Curriculum 2023.** *Am J Kidney Dis* 2023, **82:**617-634.
2. Mao W, Wu J, Zhang Z, Xu Z, Xu B, Chen M: **Neutrophil-lymphocyte ratio acts as a novel diagnostic biomarker for kidney stone prevalence and number of stones passed.** *Transl Androl Urol* 2021, **10:**77-86.
3. Turk C, Petrik A, Sarica K, Seitz C, Skolarikos A, Straub M, Knoll T: **EAU Guidelines on Interventional Treatment for Urolithiasis.** *Eur Urol* 2016, **69:**475-482.
4. Geraghty RM, Cook P, Walker V, Somani BK: **Evaluation of the economic burden of kidney stone disease in the UK: a retrospective cohort study with a mean follow-up of 19 years.** *BJU Int* 2020, **125:**586-594.
5. Hu D, Li G, Ge D, Ke L, Shu H, Chen Y, Hao Z: **The ratio of perirenal fat thickness to renal parenchymal thickness, a novel indicator of fat accumulation associated with kidney stones.** *Lipids Health Dis* 2025, **24:**86.
6. Collaborators GBDO, Afshin A, Forouzanfar MH, Reitsma MB, Sur P, Estep K, Lee A, Marczak L, Mokdad AH, Moradi-Lakeh M, et al: **Health Effects of Overweight and Obesity in 195 Countries over 25 Years.** *N Engl J Med* 2017, **377:**13-27.
7. Ward ZJ, Bleich SN, Cradock AL, Barrett JL, Giles CM, Flax C, Long MW, Gortmaker SL: **Projected U.S. State-Level Prevalence of Adult Obesity and Severe Obesity.** *N Engl J Med* 2019, **381:**2440-2450.
8. Islam MS, Wei P, Suzauddula M, Nime I, Feroz F, Acharjee M, Pan F: **The interplay of factors in metabolic syndrome: understanding its roots and complexity.** *Mol Med*

2024, **30:**279.

9. Ye Z, Wu C, Xiong Y, Zhang F, Luo J, Xu L, Wang J, Bai Y: **Obesity, metabolic dysfunction, and risk of kidney stone disease: a national cross-sectional study.** *Aging Male* 2023, **26:**2195932.

10. Saenz-Medina J, Munoz M, Rodriguez C, Sanchez A, Contreras C, Carballido-Rodriguez J, Prieto D: **Endothelial Dysfunction: An Intermediate Clinical Feature between Urolithiasis and Cardiovascular Diseases.** *Int J Mol Sci* 2022, **23**.

11. Zuo L, Tozawa K, Okada A, Yasui T, Taguchi K, Ito Y, Hirose Y, Fujii Y, Niimi K, Hamamoto S, et al: **A paracrine mechanism involving renal tubular cells, adipocytes and macrophages promotes kidney stone formation in a simulated metabolic syndrome environment.** *J Urol* 2014, **191:**1906-1912.

12. Heymsfield SB, Scherzer R, Pietrobelli A, Lewis CE, Grunfeld C: **Body mass index as a phenotypic expression of adiposity: quantitative contribution of muscularity in a population-based sample.** *Int J Obes (Lond)* 2009, **33:**1363-1373.

13. Liu Y, Wang L, Luo M, Chen N, Deng X, He J, Zhang L, Luo P, Wu J: **Inhibition of PAI-1 attenuates perirenal fat inflammation and the associated nephropathy in high-fat diet-induced obese mice.** *Am J Physiol Endocrinol Metab* 2019, **316:**E260-E267.

14. Guo XL, Wang JW, Tu M, Wang W: **Perirenal fat thickness as a superior obesity-related marker of subclinical carotid atherosclerosis in type 2 diabetes mellitus.** *Front Endocrinol (Lausanne)* 2023, **14:**1276789.

15. Ricci MA, Scavizzi M, Ministrini S, De Vuono S, Pucci G, Lupattelli G: **Morbid obesity and hypertension: The role of perirenal fat.** *J Clin Hypertens (Greenwich)* 2018, **20:**1430-1437.

16. Fang Y, Xu Y, Yang Y, Liu C, Zhao D, Ke J: **The Relationship between Perirenal Fat Thickness and Reduced Glomerular Filtration Rate in Patients with Type 2 Diabetes.** *J Diabetes Res* 2020, **2020:**6076145.

17. Hakam N, Lui JL, Shaw NM, Breyer BN: **Cushioning the blow: role of perirenal fat in renal trauma injury severity.** *BJU Int* 2023, **131:**208-212.

18. Lama DJ, Safiullah S, Yang A, Okhunov Z, Landman J, Clayman RV: **Three-dimensional evaluation of perirenal fat volume in patients with nephrolithiasis.** *Urolithiasis* 2018, **46:**535-541.

19. Davidiuk AJ, Parker AS, Thomas CS, Leibovich BC, Castle EP, Heckman MG, Custer K, Thiel DD: **Mayo adhesive probability score: an accurate image-based scoring system to predict adherent perinephric fat in partial nephrectomy.** *Eur Urol* 2014, **66:**1165-1171.

20. Peng S, Yan W, Yan Y, Tang Q, Feng H, Huang X: **AP2M1 as the potential biomarker for prediction of the response of atopic dermatitis to Dupilumab therapy: Multi-omics analysis and evidence.** *Int J Biol Macromol* 2025, **297:**139757.

21. Chen X, Zheng Z, Xie D, Xia L, Chen Y, Dong H, Feng Y: **Serum lipid metabolism characteristics and potential biomarkers in patients with unilateral sudden sensorineural hearing loss.** *Lipids Health Dis* 2024, **23:**205.

22. Chen G, Qi H, Jiang L, Sun S, Zhang J, Yu J, Liu F, Zhang Y, Du S: **Integrating single-cell RNA-Seq and machine learning to dissect tryptophan metabolism in ulcerative colitis.** *J Transl Med* 2024, **22:**1121.

23. Xu X, Pan T, Zhong X, Du Y, Zhang D: **Associations of the triglyceride-glucose index**

and remnant cholesterol levels with the prevalence of Carotid Plaque in patients with type 2 diabetes: a retrospective study. *Lipids Health Dis* 2025, **24:**26.

24. Yadegar A, Mohammadi F, Seifouri K, Mokhtarpour K, Yadegar S, Bahrami Hazaveh E, Seyedi SA, Rabizadeh S, Esteghamati A, Nakhjavani M: **Surrogate markers of insulin resistance and coronary artery disease in type 2 diabetes: U-shaped TyG association and insights from machine learning integration.** *Lipids Health Dis* 2025, **24:**96.
25. Hu C, Ge M, Liu Y, Tan W, Zhang Y, Zou M, Xiang L, Song X, Guo H: **From inflammation to depression: key biomarkers for IBD-related major depressive disorder.** *J Transl Med* 2024, **22:**997.
26. Tai Q, Xue W, Li M, Zhuo S, Zhang H, Fang F, Zhang J: **Survival Nomogram for Metastasis Colon Cancer Patients Based on SEER Database.** *Front Genet* 2022, **13:**832060.
27. Liberzon A, Birger C, Thorvaldsdottir H, Ghandi M, Mesirov JP, Tamayo P: **The Molecular Signatures Database (MSigDB) hallmark gene set collection.** *Cell Syst* 2015, **1:**417-425.
28. Yoodee S, Peerapen P, Boonmark W, Thongboonkerd V: **The inhibitory effects of proteins secreted from trigonelline-treated renal cells on calcium oxalate crystals in vitro: Implications for kidney stone prevention.** *Biomed Pharmacother* 2025, **186:**118003.
29. Ma Y, Cheng C, Jian Z, Wen J, Xiang L, Li H, Wang K, Jin X: **Risk factors for nephrolithiasis formation: an umbrella review.** *Int J Surg* 2024, **110:**5733-5744.
30. Dassanayake SN, Lafont T, Somani BK: **Association and risk of metabolic syndrome and kidney stone disease: outcomes from a systematic review and meta-analysis.** *Curr Opin Urol* 2025, **35:**377-384.
31. Huang H, Chen S, Zhang W, Wang T, Bai P, Xing J, Wang H, Chen B: **High perirenal fat thickness predicts a greater risk of recurrence in Chinese patients with unilateral nephrolithiasis.** *Ren Fail* 2023, **45:**2158870.
32. Smit RAJ, Wade KH, Hui Q, Arias JD, Yin X, Christiansen MR, Yengo L, Preuss MH, Nakabuye M, Rocheleau G, et al: **Polygenic prediction of body mass index and obesity through the life course and across ancestries.** *Nat Med* 2025.
33. Semins MJ, Shore AD, Makary MA, Magnuson T, Johns R, Matlaga BR: **The association of increasing body mass index and kidney stone disease.** *J Urol* 2010, **183:**571-575.
34. Tastemur S, Senel S, Olcucuoglu E, Uzun E: **Evaluation of the Relationship between Fat Volume and Nephrolithiasis.** *Curr Med Imaging* 2022, **18:**398-403.
35. Favre G, Grangeon-Chapon C, Raffaelli C, Francois-Chalmin F, Iannelli A, Esnault V: **Perirenal fat thickness measured with computed tomography is a reliable estimate of perirenal fat mass.** *PLoS One* 2017, **12:**e0175561.
36. Xu J, Zhou H, Cheng Y, Xiang G: **Identifying potential signatures for atherosclerosis in the context of predictive, preventive, and personalized medicine using integrative bioinformatics approaches and machine-learning strategies.** *EPMA J* 2022, **13:**433-449.
37. Belyaeva OV, Wirth SE, Boeglin WE, Karki S, Goggans KR, Wendell SG, Popov KM, Brash AR, Kedishvili NY: **Dehydrogenase reductase 9 (SDR9C4) and related homologs recognize a broad spectrum of lipid mediator oxylipins as substrates.** *J Biol Chem* 2022, **298:**101527.
38. Sriwattanapong K, Theerapanon T, Khamwachirapitak C, Sae-Ear P, Sa-Ard-Iam N,

Shotelersuk V, Porntaveetus T: **In-depth investigation of FAM20A insufficiency effects on deciduous dental pulp cells: Altered behaviours, osteogenic differentiation, and inflammatory gene expression.** *Int Endod J* 2024, **57:**745-758.

39. He J, Le Q, Wei Y, Yang L, Cai B, Liu Y, Hong B: **Effect of piperine on the mitigation of obesity associated with gut microbiota alteration.** *Curr Res Food Sci* 2022, **5:**1422-1432.
40. Krueger ABC, Zhu X, Siddiqi S, Whitehead EC, Tang H, Jordan KL, Lerman A, Lerman LO: **Mesenchymal Stem/Stromal Cells Reverse Adipose Tissue Inflammation in Pigs with Metabolic Syndrome and Renovascular Hypertension.** *Cells* 2025, **14**.
41. He K, Tang Q, Gong M, Yang S, Chen X, Zhu H, Liu D, Huang B: **A transcriptomic study of selenium against liver injury induced by beta-cypermethrin in mice by RNA-seq.** *Funct Integr Genomics* 2020, **20:**343-353.

Table 1. The study contains information regarding the datasets.

GSE series	Array type	Species	Source types	Number	Utilization
GSE94752	GPL11532	*Homo sapiens*	Abdominal subcutaneous adipocytes	30 obesity vs 9 control adipocytes	1) OB associated DEGs extraction；2) Key module gene mining via WGCNA
GSE73680	GPL17077	*Homo sapiens*	Human kidney papillary tissue biopsies	29 plaque vs 6 normal renal papillary samples	1) KSD associated DEGs extraction; 2) Hub-gene mining via ML; 3) Nomogram building
GSE117518	GPL21827	*Homo sapiens*	Human kidney papillary tissue biopsies	3 plaque vs 3 normal renal papillary samples	Validating the nomogram model

iThenticate Page 25 of 27 - Integrity Submission Submission ID trn:oid:::3117:475921701

Table 2. Baseline demographic of the study cohort (n = 413).

Variable	KSD group (n=265)	Control group (n=148)	*P*
Age (years)	54.38 ± 15.27	53.61 ± 17.69	0.646
Gender, n (%)			0.029
Male	206 (77.7%)	89 (60.1%)	
Female	59 (22.3%)	59 (39.9%)	
BMI (kg/m^2)	24.48 ± 3.33	22.45 ± 3.61	< 0.001
PFT (mm)	20.35 ± 7.69	10.98 ± 6.77	< 0.001
History of diabetes			0.04
Yes, n (%)	66 (24.9%)	24 (16.2%)	
No, n (%)	199 (75.1%)	124 (83.8%)	
History of hypertension			0.19
Yes, n (%)	90 (34.0%)	41 (27.7%)	
No, n (%)	175 (66.0%)	107 (72.3%)	
History of hyperlipidemia			0.001
Yes, n (%)	63 (23.8%)	16 (10.8%)	
No, n (%)	202 (76.2%)	132 (89.2%)	

Figure 1. Flowchart of this study.

Figure 2 PFT at the renal hilum level can be evaluated by means of transverse CT imaging. The yellow line runs from the renal capsule to the sidewall.

Figure 3. The connection between OB and KSD risk. Univariate (A) and multivariate (B) logistic regression analyses of KSD. ROC curves for evaluating BMI (C) and PFT (D) for KSD diagnosis in males and BMI (E) and PFT (F) for KSD diagnosis in females. The values of AUC (95%CI) represent the area under the curve with 95% confidence intervals.

Figure 4. Identification of OB-associated DEGs and OB-related gene modules via WGCNA. (A) Volcano plot and (B) heatmap of OB-associated DEGs between the OB

iThenticate Page 25 of 27 - Integrity Submission Submission ID trn:oid:::3117:475921701

and control groups in the GSE94752 dataset. (C) Scale independence and mean connectivity analyses for determining the optimal soft threshold power (β = 2). (D) Hierarchical clustering dendrogram of co-expressed genes, with modules color-coded. (E) Module–trait correlations: rows represent gene modules, and columns indicate clinical outcomes. Correlation coefficients (red: positive, green: negative) and *P* values (in parentheses) are shown. (F, G) Scatter plots of module membership versus gene significance for KSD in the turquoise (F) and yellow (G) modules. Genes meeting the threshold (module membership > 0.8, gene significance > 0.5) were prioritized as key module genes.

Figure 5. Identification of KSD-associated DEGs and OB-related DEGs (ORDEGs). (A) Volcano plot and (B) heatmap of KSD-associated DEGs between the KSD and control groups in the GSE73680 dataset. (C) Venn diagram showing the overlap among OB-associated DEGs, KSD-associated DEGs, and key module genes. The intersecting genes were identified as ORDEGs. (D) Box plot illustrating the expression patterns of the eight candidate ORDEGs.

Figure 6. Identification of hub ORDEGs associated with KSD via machine learning approaches. (A, B) LASSO regression analysis of the eight candidate ORDEGs. (C) RF classification tree. (D) Variable importance ranking based on the Gini index from the RF algorithm. (E, F) Optimal feature selection of ORDEGs via the SVM-RFE algorithm. (G) Venn diagram showing consensus genes from three methods: the top 5 genes from random forest, the 4 genes from LASSO regression, and the 7 feature genes from SVM-RFE analysis.

Figure 7. Development and validation of the diagnostic nomogram model for KSD. (A) Nomogram constructed on the basis of the two hub genes. (B) Calibration curve assessing the agreement between the predicted and observed KSD probabilities. (C) DCA curve evaluating the clinical utility of the nomogram. (D-E) ROC curves demonstrating the model's diagnostic performance in the (D) internal GSE73680 dataset and (E) external validation GSE117518 dataset from GEO.

Figure 8. Pathway enrichment of the two hub genes. Pathway enrichment analysis of hub genes (FAM20A/DHRS9), showing significant correlation with inflammatory and metabolic pathways (e.g., IL-6/JAK/STAT3, TNF-α/NF-κB). *$P < 0.05$, **$P < 0.01$, ***$P < 0.001$, ns, $P \geq 0.05$.
